# Semiconducting Open‐Shell Radicals for Precise Tumor Activatable Phototheranostics

**DOI:** 10.1002/advs.202500293

**Published:** 2025-03-07

**Authors:** Jie Zhang, Haifen Luo, Wen Ma, Jingqi Lv, Bo Wang, Fengwei Sun, Weijie Chi, Zhuting Fang, Zhen Yang

**Affiliations:** ^1^ Strait Laboratory of Flexible Electronics (SLoFE) Fujian Key Laboratory of Flexible Electronics Strait Institute of Flexible Electronics (Future Technologies) Fujian Normal University Fuzhou 350117 China; ^2^ Department of Oncology and Vascuar Interventonal Therapy Clinical Oncology School of Fujian Medical University Fujian Cancer Hospital Fuzhou 350014 China; ^3^ Department of imterventional Radiology shengli Clinical Medical College of Fujian Medical University Fuijian Provincial Hospital Fuzhou University Affliated Provincial Hospital Fuzhou 350001 China; ^4^ School of Chemistry and Chemical Engineering Hainan University Haikou 570228 China

**Keywords:** CDT, immunotherapy, NIR‐II FLI, semiconducting open‐shell radicals, tumor activatable phototheranostics, type‐I PDT

## Abstract

Semiconducting open‐shell radicals (SORs) have promising potential for the development of phototheranostic agents, enabling tumor bioimaging and boosting tumorous reactive oxygen species (ROS). Herein, a new class of semiconducting perylene diimide (PDI), designated as PDI(Br)_n_ with various numbers of bromine (Br) atoms modified on PDI's bay/ortho positions is reported. PDI(Br)_n_ is demonstrated to transform into a radical anion, [PDI(Br)_n_]^•−^, in a reducing solution, with a typical *g*‐value of 2.0022. Specifically, [PDI(Br)_4/6_]^•−^ is generated in the weakly reductive tumor‐mimicking solution and exhibits high stability in air. Quantum chemical kinetic simulation and ultrafast femtosecond transient absorption spectroscopy indicate that [PDI(Br)_6_]^•−^ has a low *π–π* stacking energy (0.35 eV), a fast electron transfer rate (192.4 ps) and energy gap of PDI(Br)_6_ (Δ*E*_S1, T1 _= 1.307 eV, Δ*E*_S1, T2 _= 0.324 eV) respectively, which together result in excited‐state charge transfer characters. The PDI(Br)_6_ nanoparticle radicals, [PDI(Br)_6_] NPs^•−^, specifically enable chemodynamic and type‐I photodynamic ROS generation in tumors, including superoxide and hydroxyl radicals, which elicit immunogenic cell death effect. Also, [PDI(Br)_6_] NPs^•−^ facilitate activatable bioimaging‐guided therapy due to their photoacoustic signal at 808 nm and NIR‐II emission at 1115 nm. The work paves the way for the design of SORs for precise cancer theranostics.

## Graphical abstract

A versatile phototheranostics nanoplatform of tumor‐specific activatable semiconducting open‐shell radicals for precise chemodynamic/photodynamic therapy (CDT/PDT) guided by “Turn On” second near‐infrared (NIR‐II) window fluorescence imaging (FLI) and PA imaging (PAI) through molecular engineering strategies.

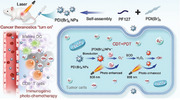

## 1 Introduction

Cancer phototheranostics, integrating photodiagnosis and phototherapy with the assistance of biophotonic agents, enables the treatment with noninvasiveness, spatial localization, and manipulability.^[^ ^1^, ^2^, ^3^, ^4^ ^]^ To achieve precision phototheranostics, the ideal photonic molecules are required to possess activatable optical and pharmacological “turn on” properties in response to the specific tumor microenvironment (TME), such as excessive glutathione (GSH), reactive oxygen species (ROS), cytochrome reductase, nitro‐reductase, to achieve highly sensitive differentiation of tumor by precise imaging with enhanced optical imaging contrast, simultaneously provide highly efficient therapeutic efficacy with minimized side effects.^[^ ^5^, ^6^, ^7^, ^8^, ^9^ ^]^ Type‐I photodynamic therapy (Type‐I PDT) as a promising approach has been explored for cancer phototheranostics, owing to the generation of photoinduced cytotoxic long‐lived ROS, such as superoxide radicals (O_2_^•−^) and hydroxyl radicals (•OH), beyond the Type‐II PDT with the generation of short‐lived singlet oxygen (^1^O_2_).^[^ ^10^, ^11^, ^12^, ^13^, ^14^, ^15^ ^]^ The ROS‐specific generation in cancer cells further results in cellular immunogenic cell death (ICD), priming the cancer immunotherapy and reducing the side effects of phototherapy.^[^ ^16^, ^17^ ^]^ Type‐I PDT is O_2_ less dependent, especially due to the O_2_ compensation in hypoxic cancer cells by O_2_^•−^, which undergoes Haber–Weiss reactions or intratumoral disproportionation.^[^ ^18^, ^19^ ^]^ By taking account of specific TME, the activable Type‐I PDT agent guided by “turn on” imaging modalities is highly required to achieve precision phototheranostics.^[^ ^20^, ^21^, ^22^, ^23^, ^24^, ^25^ ^]^

Semiconducting open‐shell radicals (SORs) owning *π*‐conjugated skeleton, unpaired electrons in their singly occupied molecular orbital (SOMO), narrow optical band gaps, and biocompatibility show strong intermolecular stacking, long‐wavelength absorption/emission, and high photothermal conversion, which have potential for cancer phototheranostics.^[^ ^26^, ^27^, ^28^, ^29^, ^30^, ^31^, ^32^ ^]^ Recently, semiconducting closed‐shell materials (SCMs) including tetrathiafulvalene, perylene/naphthalene diimide, bipyridine, and *π‐*extended nickel corrole have been modified into their SORs as photothermal agents showing potential for cancer phototheranostics.^[^ ^33^, ^34^, ^35^, ^36^, ^37^, ^38^ ^]^ The majority of the SORs are synthesized by their original SCMs receiving electrons from strong reductants and sacrificial electron donors.^[^ ^39^, ^40^ ^]^ The resulting SORs are usually short‐lived and readily return to their SCMs when exposed to air or a physiological environment.^[^ ^41^, ^42^ ^]^ The SORs also tend to favor non‐radiative energy release leading to weak fluorescence emission and almost no photodynamic properties, which greatly hinders their further bioapplications of photo theranostics.^[^ ^43^, ^44^, ^45^ ^]^

**Figure 1:**
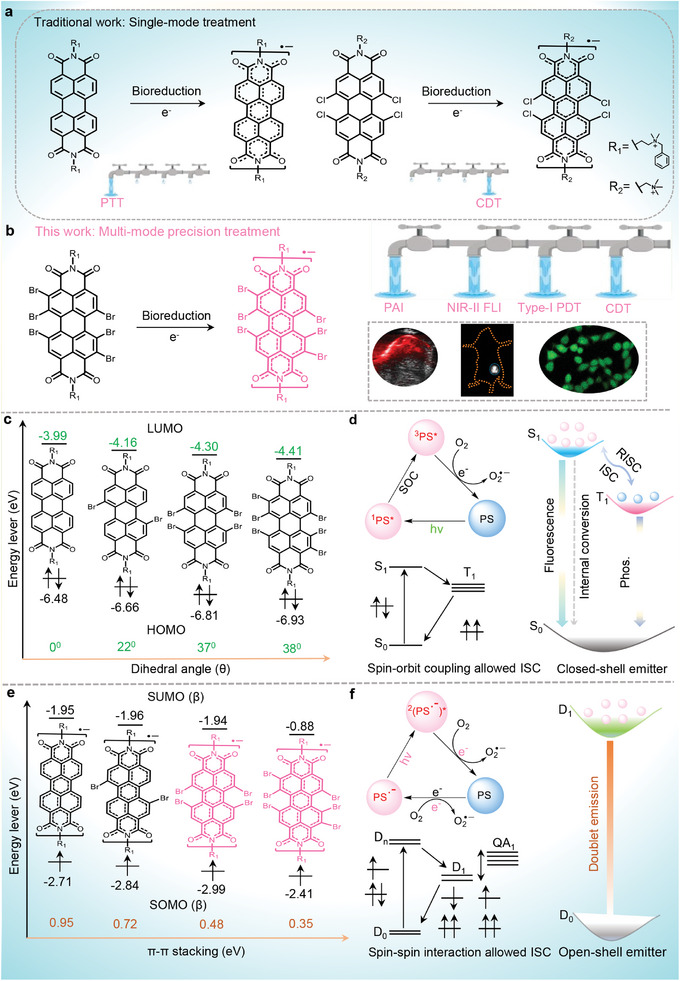
Traditional single‐mode treatment, multi‐mode precision treatment, and photophysical processes involved in Type‐I PDT and luminescence for PDI semiconducting closed‐shell and open‐shell radicals. a) The single‐model treatment (traditional strategies)^[^ ^46^, ^47^, ^60^ ^]^ and b) multi‐mode precision treatment for cancer of PDI semiconducting open‐shell radicals (this work). c,d) The chemical structures, energy levels, dihedral angle, spin–orbit coupling allowed ISC and luminescence mechanism of PDI closed‐shell materials.^[^ ^64^, ^65^, ^66^ ^]^ e,f) The chemical structures, energy levels, *π–π* stacking energy levels, spin–spin interaction allowed ISC and luminescence mechanism of PDI open‐shell radicals.^[^ ^39^, ^67^, ^68^, ^69^ ^]^

**Scheme 1:**
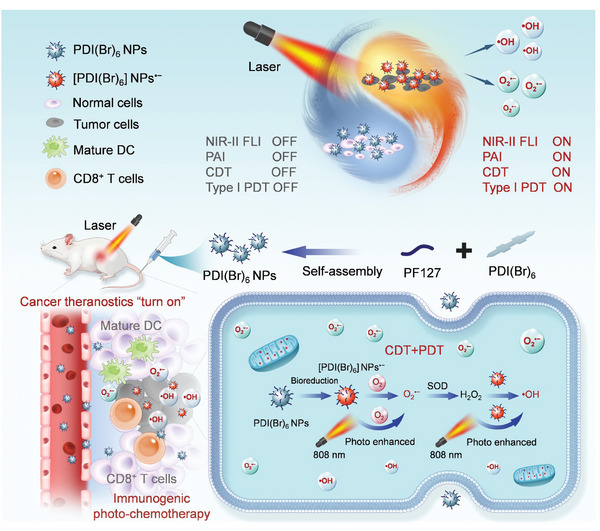
Schematic of a cancer theranostic nanoplatform of activatable PDI semiconducting open‐shell radicals (PDI SORs) for combined chemodynamic/photodynamic therapy (CDT/PDT) guided by NIR‐II fluorescence imaging (FLI) and PA imaging (PAI).

To improve the photophysical properties of the SORs to meet the needs of advanced phototheranostics, especially the PDT and fluorescence imaging (FLI) in the second near‐infrared region (NIR‐II, 1000–1700 nm), we investigate a series of semiconducting perylene diimide (PDI) based SORs with various bromo (Br)‐substituents [PDI(Br)_n_]^•−^ on the PDI bay/ortho positions (**Figure** **1**). According to theoretical calculations, the dihedral angle of aggregated [PDI(Br)_n_]^•−^ is enlarged from 0° to 38°, increasing the even number of Br atoms from 0 to 6. Thus, we hypothesize that the introduction of Br groups enhances the PDT and FL properties of [PDI(Br)_n_]^•−^, due to the increased heavy atoms effect and weakened *π–π* stacking energy (from 0.95 to 0.35 eV). Additionally, owing to the electron‐drawing effect of Br atoms, the reduction potential of PDI(Br)_n_ decreases gradually from −402 to −203 mV, and its orbital energy level shifts from −3.99 to −4.41 eV. The [PDI(Br)_n_]^•−^ tends to be more stable and more easily obtained in the reducing TME with the increase of Br‐substituents. Thus, in this work, the SCMs (PDI(Br)_6_) with six Br‐substituents on the PDI core have been demonstrated easy to get electrons from reducing solutions with mimicking of TME to form SORs ([PDI(Br)_6_]^•−^), which is quite stable in the air. After encapsulation by amphiphilic polymer F‐127, PDI(Br)_6_ based nanoparticles (PDI(Br)_6_ NPs) specifically transform into radical anion [PDI(Br)_6_] NPs^•−^ in tumor cells or solid tumors (**Scheme** **1**). Due to the rising 808 nm absorbance and NIR‐II emission (peak at 1115 nm), [PDI(Br)_6_] NPs^•−^ enable the tumor‐specific “turn on” photoacoustic imaging (PAI) and NIR‐II FLI. Intriguing, the [PDI(Br)_6_] NPs^•−^ exhibit a fast electron transfer rate (192.4 ps), which contributes to their catalytic performance for tumor chemodynamic therapy (CDT) by turning O_2_ and H_2_O_2_ into O_2_^•−^ and •OH, respectively.^[^ ^46^, ^47^ ^]^ Furthermore, [PDI(Br)_6_] NPs^•−^ show Type‐I PDT property of enhanced ROS generation upon 808 laser irradiation. Thus, the PDI(Br)_6_ NPs achieve tumor‐targeted phototherapeutic with minimized side effects and high phototherapeutic efficacy for inhibiting tumor growth. The investigation of activatable PDI‐based SORs paves a new avenue of cancer phototheranostics in clinical applications.

## 2 Results

### 2.1 Molecular Design, Synthesis and Characterization

**Figure 2:**
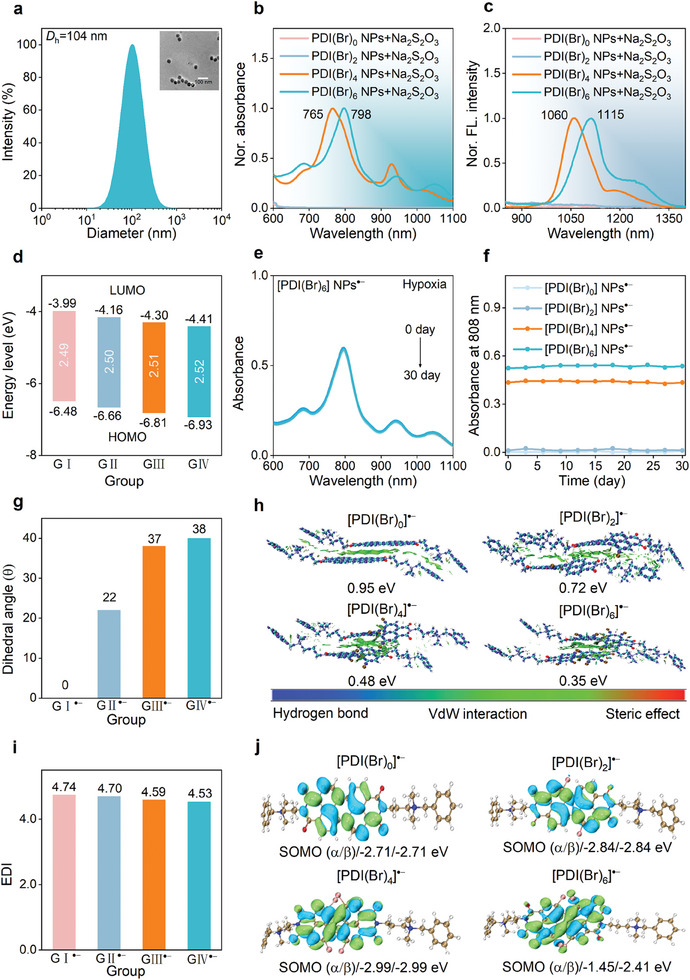
Photophysical properties studies of the molecular PDI(Br)_n_ and nanoparticle PDI(Br)_n_ NPs and their corresponding radicals. a) DLS analysis of PDI(Br)_6_ NPs. Inset: TEM image of PDI(Br)_6_ NPs. b) Normalized absorption and c) NIR‐II fluorescence spectra of PDI(Br)_n_ NPs (0.2 mm, 2.5 mL) obtained by titration with Na_2_S_2_O_3_ solution. d) The calculated HOMO, LUMO, and energy levels of the four kinds of PDI(Br)_0/2/4/6_. e) After reaction with Na_2_S_2_O_3_, time‐dependent absorption spectra of [PDI(Br)_6_] NPs^•−^ and f) comparison of the absorption stability of four radials [PDI(Br)_n_] NPs^•−^ recording at 808 nm during 30 days in PBS under hypoxia conditions. g) The calculated dihedral angle distribution and h) simulated interaction modes and interaction energies of the four kinds of [PDI(Br)_n_]^•−^ in their aggregates, respectively. i) The calculated electron delocalization index levels and j) molecular orbital energy levels of the four kinds of [PDI(Br)_n_]^•−^. GI/GI^•−^: PDI(Br)_0_/[PDI(Br)_0_]^•−^, GII/GII^•−^: PDI(Br)_2_/[PDI(Br)_2_]^•−^, GIII/GIII^•−^: PDI(Br)_4_/[PDI(Br)_4_]^•−^, GIV/GIV^•−^: PDI(Br)_6_/[PDI(Br)_6_]^•−^.

Based on the PDI scaffold, four PDI derivatives PDI(Br)_0/2/4/6_ modified with various numbers of Br atoms were synthesized through bromination reaction (Schemes S1–S4, Supporting Information).^[^ ^48^, ^49^ ^]^ The structures of the intermediates and the target molecules were fully characterized by nuclear magnetic resonance spectroscopy and high‐resolution mass spectrometry (HRMS) (Figures S1–S15, Supporting Information). To obtain biocompatible agents, the hydrophobic PDI(Br)_0/2/4/6_ were encapsulated into water‐soluble NPs with nanoprecipitation method by using amphiphilic polymer Pluronic F‐127 as the surfactant. Dynamic light scattering (DLS) and transmission electron microscopy (TEM) analyses confirmed that PDI(Br)_6_ NPs exhibited a uniformly spherical morphology with an average hydrodynamic diameter of 104 nm, consistent with that of PDI(Br)_0/2/4_ NPs. The four molecules PDI(Br)_0/2/4/6_ showed a positive charge in solution, and after encapsulation, PDI(Br)_0/2/4/6_ NPs showed negative zeta potential, which demonstrated that the four kinds of PDI were well encapsulated by PF‐127 (**Figure** **2**a; Figures S16,S17, Supporting Information). The fabrication of nanoparticles allowed PDIs to passively aggregate at tumor sites through enhanced permeability and retention effects. The diameter changes of these NPs were negligible during 14 days’ storage in PBS solution supplemented with 10% fetal bovine serum, indicating excellent stability (Figure S18, Supporting Information).

Subsequently, the photophysical properties of four kinds of PDI NPs were investigated by measuring their absorption and fluorescence spectra. The absorption maximum peaks of the PDI(Br)_0/2/4/6_ NPs between 500–600 nm, and the maximum emission peaks were between 550–650 nm (Figures S19,S20, Supporting Information), indicating that the NPs were difficult to carry out NIR‐II imaging and therapy in vivo.^[^ ^50^, ^51^ ^]^ We then investigated the potential of PDI SORs by reduction of PDI(Br)_0/2/4/6_ for cancer theranostics.^[^ ^52^ ^]^ In this work, strong reducing agent sodium dithionate (Na_2_S_2_O_4_) and weak reducing agent sodium thiosulfate (Na_2_S_2_O_3_) were utilized as to explore the generation of PDI SORs.^[^ ^53^ ^]^ After reacting with Na_2_S_2_O_4_, the absorption of the four kinds of PDI NPs all displayed new peaks at 600–1100 nm, indicating the formation of PDI SORs. However, when Na_2_S_2_O_3_ solution was introduced, only PDI(Br)_4_ NPs and PDI(Br)_6_ NPs were reduced to the corresponding SORs showing the maximum absorption peak at 765 and 798 nm, respectively (Figure 2b; Figures S21,S22, Supporting Information). Additionally, the first reduction peak of PDI(Br)_0_, PDI(Br)_2_, PDI(Br)_4_, and PDI(Br)_6_ were observed at −402, −347, −289, and −203 mV versus Ag/AgCl in solutions, respectively (Figure S23, Supporting Information). The lowest unoccupied molecular orbitals (LUMOs) and the highest occupied molecular orbitals (HOMOs) of these compounds were primarily distributed along the PDI molecular backbone. Meanwhile, the theoretical calculations estimated LUMO values of −3.99, −4.16, −4.3, and −4.41 eV for the four PDIs, respectively (Figure 2d; Figures S24,S25, Supporting Information), demonstrating that PDI(Br)_4_ NPs and PDI(Br)_6_ NPs were more easily to transform into the SORs [PDI(Br)_4_] NPs^•−^ and [PDI(Br)_6_] NPs^•−^ under mild reduction environment, such as TME.

We then investigated the above NIR‐II fluorescence emission of the four kinds of PDI NPs after the reaction with Na_2_S_2_O_4_ and Na_2_S_2_O_3_ by using 808 nm laser irradiation. After reaction with Na_2_S_2_O_4_, PDI(Br)_0_ NPs showed barely NIR‐II emission, the other three kinds of PDI NPs exhibited strong NIR‐II emission in the range of 900–1400 nm. However, under reaction with Na_2_S_2_O_3_, PDI(Br)_0_ NPs and PDI(Br)_2_ NPs both showed no NIR‐II fluorescence emission, and PDI(Br)_4_ NPs and PDI(Br)_6_ maintained intensive NIR‐II fluorescence emission (Figure 2c; Figures S26,S27, Supporting Information). The absence of NIR‐II fluorescence emission was likely attributed to the tendency *π–π* stacking of PDI(Br)_0_ nanoparticles in water, leading to the fluorescence quenching of SORs. Subsequently, we tested the stability performance of the generated PDI SOCs. [PDI(Br)_4_] NPs^•−^ and [PDI(Br)_6_] NPs^•−^ produced from Na_2_S_2_O_3_, exhibited prolonged photophysical stability under hypoxic conditions and across various environments, including different pH levels, serum solutions, and photothermal stability (Figure 2e,f; Figures S28–S33, Supporting Information).

To further explore the mechanism of the bright NIR‐II fluorescence emission and excellent stability of [PDI(Br)_4_] NPs^•−^ and [PDI(Br)_6_] NPs^•−^, various molecular properties, including the dihedral angle, interaction mode, electron delocalization index (EDI) and singly unoccupied molecular orbital of the four PDI(Br)_0/2/4/6_ were analyzed at the U‐B3LYP/Def2SVP level. The dihedral angle between the [PDI]^•−^ was calculated to be 38°, 37°, 22°, and 0° for [PDI(Br)_6_]^•−^, [PDI(Br)_4_]^•−^, [PDI(Br)_2_]^•−^ and [PDI(Br)0]•−, respectively (Figure 2g; Figures S34,S35, Supporting Information). These results indicate that the introduction of bromine (Br) atoms restricts *π–π* stacking between [PDIs]•− molecules, thereby enhancing the stability of the PDI SORs. To further analyze the superiorities of twisted molecule strategy, quantum chemical molecular dynamics simulations were performed on four [PDIs]^•−^. Clearly, [PDI(Br)_4_]^•−^ and [PDI(Br)_6_]^•−^ consistently maintained a twisted conformation, whether in a single‐molecule state or aggregate state. The *π–π* stacking energy of the four [PDI(Br)_0/2/4/6_]^•−^ decreased gradually from 0.95 to 0.35 eV (Figure 2h). These findings supported the importance of the twisted molecular structure in stabilizing SORs and facilitating NIR‐II fluorescence emission. A decrease in both EDI and the energy of the SOMOs on the α and β orbitals was observed, with values ranging from 4.74 to 4.53 and −2.71/−2.71 to −1.45/−2.41 eV, respectively (Figure 2i,j; Figure S36, Supporting Information), indicating reduced reactivity at a single atom and overall enhanced stability of the molecule due to spin delocalization within the large *π*‐system.^[^ ^54^ ^]^

### 2.2 Chemo/Photodynamic Properties of PDI SORs

**Figure 3:**
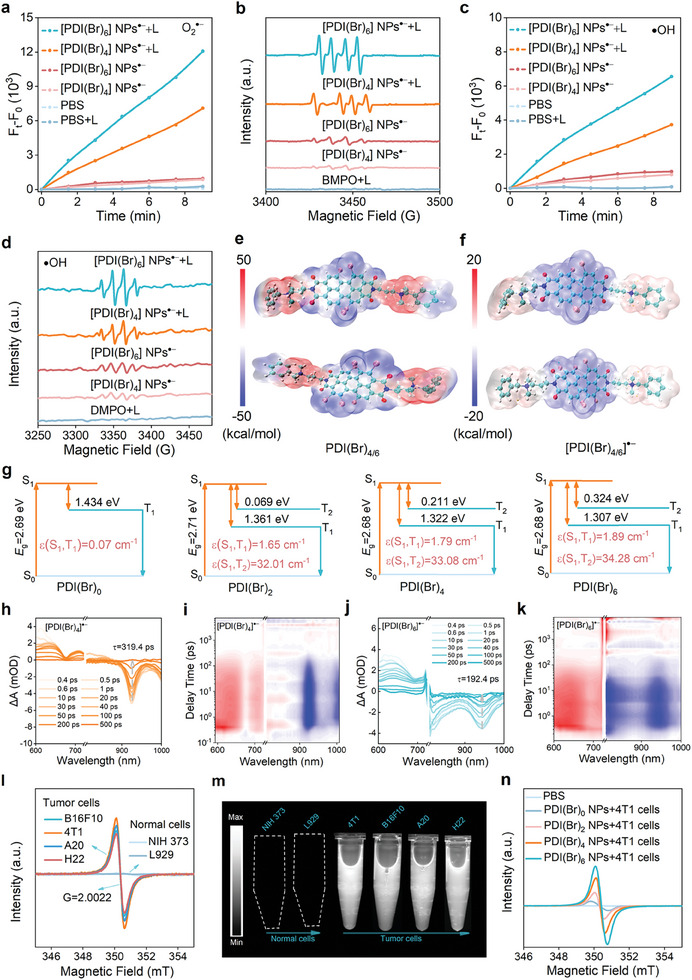
ROS performance, electrostatic potential (ESP), ultrafast femtosecond transient absorption (fs‐TA), and electron spin resonance (ESR) spectra of [PDI(Br)_n_] NPs^•−^. a) Comparison of the O_2_^•−^ generation capacity of [PDI(Br)_4_] NPs^•−^ and [PDI(Br)_6_] NPs^•−^ with or without laser irradiation (0.3 W cm^−2^ at 808 nm). b) ESR signals of BMPO for O_2_^•−^ characterization in the presence of [PDI(Br)_4_] NPs^•−^ and [PDI(Br)_6_] NPs^•−^ containing BMPO (DMSO: H_2_O = 1: 9, v/v) before and after 808 nm laser for 10 min. [BMPO] = 0.15 m. c) Comparison of the •OH generation capacity of [PDI(Br)_4_] NPs^•−^ and [PDI(Br)_6_] NPs^•−^ with or without laser irradiation (0.3 W cm^−2^ at 808 nm). H_2_O_2_ (0.2 mm) was added to all test solutions. d) ESR signals of DMPO for •OH characterization in the presence of [PDI(Br)_4_] NPs^•−^ and [PDI(Br)_6_] NPs^•−^ containing DMPO (DMSO: H_2_O = 1: 9, v/v) before and after 808 nm laser for 10 min. [DMPO] = 0.15 m. H_2_O_2_ (0.2 mm) was added to all the test solutions to obtain •OH. e) Electrostatic potential (ESP) distributions of PDI(Br)_4_ (top), PDI(Br)_6_ (bottom), f) [PDI(Br)_4_]^•−^ (top) and [PDI(Br)_6_]^•−^ (bottom), respectively. g) The energy level diagrams and SOC coefficients for the four kinds of PDI(Br)_n_. The femtosecond transient absorption spectra of h,i) [PDI(Br)_4_]^•−^ and j,k) [PDI(Br)_6_]^•−^ with a 750 nm photoirradiation at different pump‐probe delay times. l) ESR spectra and m) NIR‐II FLI of the [PDI(Br)_6_]^•−^ generated from PDI(Br)_6_ NPs in the presence of various tumor cells under hypoxic conditions and normal cells under normoxic conditions. n) ESR spectrum of the four kinds of PDI(Br)_n_ NPs incubating with 4T1 tumor cells.

Encouraged by the remarkable stability and optical properties of [PDI(Br)_4/6_] NPs^•−^, we further investigated their photodynamic and chemodynamic electron transfer performance for the generation of ROS, such as O_2_^•−^ and •OH, by using an 808 nm laser (Figure S37, Supporting Information).^[^ ^47^ ^]^ The overall ROS generation capabilities were evaluated using ROS‐sensitive dichlorodihydrofluorescein (DCFH) as a generation indicator. The fluorescence at 525 nm showed a slow increase in the presence of [PDI(Br)_4_] NPs^•−^ and [PDI(Br)_6_] NPs^•−^, respectively. However, a significant fluorescence increase was observed upon 808 nm (0.3 W cm^−2^) laser irradiation upon above the solutions (Figures S38–S40, Supporting Information). The comparison of the ROS‐producing capacity of the experimental groups was PBS < [PDI(Br)_4_] NPs^•−^ < [PDI(Br)_6_] NPs^•−^ < [PDI(Br)_4_] NPs^•− ^+ laser < [PDI(Br)_6_] NPs^•− ^+ laser. To distinguish the specific ROS species in the system, we utilized dihydrorhodamine 123 (DHR123) to detect the generation of O_2_^•−^. In the absence of laser irradiation, both [PDI(Br)_6_] NPs^•−^ and [PDI(Br)_4_] NPs^•−^ exhibited a slight fluorescence intensity of DHR123 compared to the PBS group. Surprisingly, upon near‐infrared (NIR) light irradiation, the fluorescence intensity of DHR123 in both solutions was significantly enhanced. The results indicated that both [PDI(Br)_6_] NPs^•−^ and [PDI(Br)_4_] NPs^•−^ showed CDT and Type‐I PDT abilities (**Figure** **3**a; Figures S41–S43, Supporting Information). To accurately determine the production of O_2_^•−^, ESR spectroscopy was performed using 5‐tert‐butoxycarbonyl‐5‐methyl‐1‐pyrroline N‐oxide (BMPO) as a spin‐trapping reagent. Without laser irradiation, slight O_2_^•−^ signals were detected by the ESR in solutions of [PDI(Br)_6_] NPs^•−^ and [PDI(Br)_4_] NPs^•−^. Moreover, enhanced ESR signals were observed upon laser irradiation in above the solutions (Figure 3b). To further investigate whether [PDI(Br)_6_] NPs^•−^ and [PDI(Br)_4_] NPs^•−^ produced •OH in the H_2_O_2_ solution, •OH‐specific probes hydroxyphenyl fluorescein (HPF) were employed in experiments. The [PDI(Br)_6_] NPs^•−^ and [PDI(Br)_4_] NPs^•−^ exhibited moderate •OH generation abilities without laser. Under laser irradiation, the production amount of •OH was improved. Notably, [PDI(Br)_6_] NPs^•−^ substantially increased the fluorescence intensity of HPF by 7.81‐fold compared to PBS and 1.67‐fold compared to [PDI(Br)_4_] NPs^•−^ (Figure 3c; Figures S44,S45, Supporting Information). The generation of •OH was also confirmed by ESR analysis (Figure 3d), using DMPO as spin trapping agents. Without laser irradiation, the slight •OH signals were detected in the ESR spectrum for [PDI(Br)_4_] NPs^•−^ and [PDI(Br)_6_] NPs^•−^. In contrast, enhanced ESR signals were observed upon laser irradiation. Singlet oxygen (^1^O_2_) signals of the two radicals were almost silent under the same experimental conditions (Figure S46, Supporting Information). All in all, both [PDI(Br)_6_] NPs^•−^ and [PDI(Br)_4_] NPs^•−^ could boost •OH and O_2_^•−^ production, which beneficial for cancer CDT/PDT combination therapy.

To comprehensively understand the ROS‐generation mechanism of the SORs, theoretical calculations were performed by capitalizing on time‐dependent density functional theory. The electron density diagrams illustrated an increase in electron density from PDI to PDI^•−^ (Figure 3e,f; Figures S47,S48, Supporting Information). The electron distribution on the surface of PDI^•−^ was notably high, facilitating the direct sensitization of oxygen O_2_ to produce O_2_^•−^. Upon light irradiation, PDI^•−^ was excited to the doublet excited state, ^2^(PDI^•−^)^*^, which enhanced electron transfer to O_2_, resulting in significant O_2_^•−^ generation.^[^ ^55^, ^56^ ^]^ Due to the immaturity of intersystem crossing (ISC) process calculation on SORs, thus we investigated the ISC process of PDI SCMs as lateral evidence to demonstrate the enhancement of photoelectronic process with an increase of Br atoms on the PDI skeleton. With Br substituents from 0 to 6, PDIs showed a decreased energy gap Δ*E*_ST_ from 1.434 to 1.307 eV. The spin–orbit coupling constants ξ of PDI(Br)_6_ even reached *S*_1_, *T*_1 _= 1.89 cm^−1^, *S*_1_, *T*_2 _= 34.28 cm^−1^, showing a highly enhanced ISC pathway for promoting photoelectronic transfer (Figure 3g). Strengthening the above‐proposed mechanism, the fs‐TA spectroscopy of [PDI(Br)_4_] ^•−^ and [PDI(Br)_6_]^•−^ were obtained by exciting at its peak absorption (750 nm). The positive absorption from 600 and 750 nm corresponds to the excited state absorption of ^2^(PDIs^•−^)*, and the negative absorption at 900–1000 nm corresponds to the ground state bleach (GSB) of PDI^•−^ (Figure 3h,j).^[^ ^56^, ^57^ ^]^ We quantitatively analyzed the GSB rates of [PDI(Br)_4_]^•−^ and [PDI(Br)_6_]^•−^. The results showed that the average lifetime of [PDI(Br)_6_]^•−^ (192.4 ps) is shorter than that of [PDI(Br)_4_] ^•−^ (319.4 ps), further indicated that [PDI(Br)_6_]^•−^ had more efficient electron transfer (Figure S49, Supporting Information).^[^ ^58^ ^]^ Results also were revealed from the 2D pseudocolor fs‐TA spectra that the GSB signal of [PDI(Br)_6_]^•−^ exhibited relatively fast attenuation (Figure 3i,k).^[^ ^59^ ^]^ Overall, the rapid electron transfer process within the [PDI(Br)_6_]^•−^ under laser exposure further amplified ROS production efficiency, particularly favoring the Type‐I PDT pathway.

Hypoxic tumors generally exhibit strong reducibility due to the high accumulation of reducing substances such as reducing complexes (GSH), reducing enzymes (GSH reductase), reducing amino acids (cysteine), and nicotinamide adenine dinucleotide phosphate NAD(P)H.^[^ ^60^, ^61^ ^]^ To investigate the conversion of PDI NPs into PDI NPs^•−^ in hypoxic tumor cells, ESR spectroscopy and NIR‐II fluorescent images were conducted. As shown in Figure 3l,m and Figure S50 (Supporting Information), the ESR and NIR‐II fluorescence signals were observed in the presence of various tumor cells under hypoxic conditions, suggesting the formation of [PDI(Br)_6_] NPs^•−^. In contrast, there were no ESR and fluorescence signals in the presence of normal cells under normoxic conditions. We then compared the ESR signals of the four PDI(Br)_0/2/4/6_ NPs after incubation with 4T1 tumor cells, indicating that increased ESR signals by the increase of the Br atoms on the PDI skeleton (Figure 3n). Although the PDI(Br)_0/2_ NPs showed ESR signals with 4T1 tumor cells, the signals were quenched in the 7 days of exposure in the air, but the signals of PDI(Br)_4/6_ NPs were enduring in the same condition (Figure S51, Supporting Information). Further, the spin density calculations of the four kinds of PDI SORs revealed that the single electron were delocalized across the whole PDI skeleton. Specifically, incorporating of Br atoms into the PDI enhanced electron delocalization within the intramolecular bonds of SORs, which was beneficial to reducing the reactivity on a single atom, improving the molecule's overall stability (Figures S52,S53, Supporting Information). In conclusion, the excellent photostability, ROS generation ability, and hypoxic tumor‐specific activation of PDI(Br)_6_ NPs allowed to further biomedical applications.

### 2.3 ROS Performance of PDI(Br)_6_ NPs on the Cellular Level

**Figure 4:**
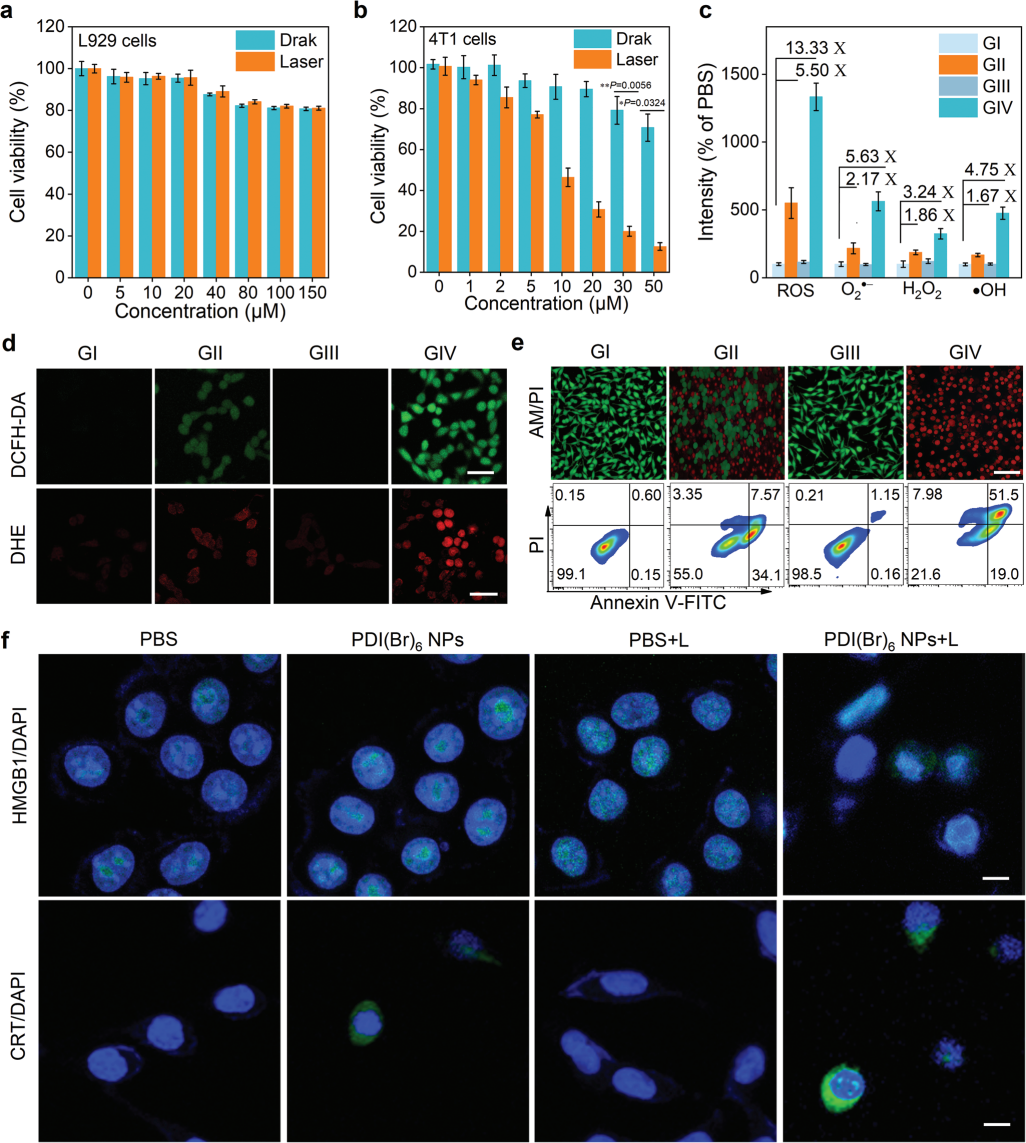
In vitro cellular research on the phototheranostics of PDI(Br)_6_ NPs. Cell viabilities of a) L929 cells and b) 4T1 cells after treatment of PDI(Br)_6_ NPs with or without laser irradiation (0.3 W cm^−2^ at 808 nm). Data are presented as mean ± SD (*n* = 4 independent experiments). c) Quantitative fluorescence analysis of total ROS, O_2_^•−^, H_2_O_2_ and •OH in 4T1 cells after treatment of PBS and PDI(Br)_6_ NPs for 12 h with or without laser irradiation (0.3 W cm^−2^ at 808 nm). Data are presented as mean ± SD (*n* = 3 independent experiments). d) Representative imaging of DCFH‐DA (ROS), dihydroethidium (DHE) (O_2_^•−^) in 4T1 cells treated with PBS or PDI(Br)_6_ NPs for 12 h with or without laser irradiation (0.3 W cm^−2^ at 808 nm). Scale bars: 50 µm. e) Live/dead cells staining (top) and cell apoptosis assay (bottom) of 4T1 cells after various treatments, Scale bars: 50 µm. f) Representative images of the HMGB1 release (top) and CRT exposure (bottom) on 4T1 cancer cells after various treatments. Scale bars: 40 µm. GI: PBS, GII: PDI(Br)_6_ NPs, GIII: PBS+laser, GIV: PDI(Br)_6_ NPs+laser.

Based on the CDT/PDT performance, we first investigated the cytotoxicity of PDI(Br)_6_ NPs on L929 cells and 4T1 cancer cells by cell counting kit‐8 (CCK‐8) assays. Results demonstrated that PDI(Br)_6_ NPs showed negligible cytotoxicity with or without 808 nm laser irradiation on L929 cells, indicating it had good biocompatibility on normal cells. However, obvious 4T1 cell death was observed with the treatment of PDI(Br)_6_ NPs with or without 808 nm laser (**Figure** **4**a,b). The half‐maximal inhibitory concentration (IC50) of PDI(Br)_6_ NPs was less than 10 µm under laser in the hypoxic environment, indicating that PDI(Br)_6_ NPs were specifically activated in hypoxic tumor cells with excellent CDT and Type‐I PDT properties. DCFH‐DA was used to evaluate the intracellular ROS‐producing capability of PDI(Br)_6_ NPs by confocal laser scanning microscopy and flow cytometry. When 4T1 cells were treated exclusively with PDI(Br)_6_ NPs, green signals associated with ROS were observed, suggesting that the PDI(Br)_6_ NPs were activated into [PDI(Br)_6_] NPs^•−^ and then induced endogenous ROS generation under hypoxic conditions. After laser irradiation, strong green fluorescence signals were observed, revealing the PDT of [PDI(Br)_6_] NPs^•−^ in 4T1 cells performed (Figure 4d, Top). The same results were obtained by flow cytometry (Figure S54, Supporting Information). Subsequently, intracellular O_2_^•−^ generation was detected by dihydroethidium (DHE) fluorescent probe. Red fluorescence signals were observed in the PDI(Br)_6_ NPs‐treated 4T1 cells under hypoxic conditions, and the signals became brighter after laser irradiation (Figure 4d, Bottom), indicating the O_2_^•−^ is the main component of the generated endogenous ROS. As known, O_2_^•−^ can trigger a series of reactions to the production of H_2_O_2_ and O_2_.^[^ ^47^, ^62^, ^63^ ^]^ Additionally, [PDI(Br)_6_] NPs^•−^ reacted with H_2_O_2_ to produce •OH. Thus, we used commercial H_2_O_2_ and •OH probes to quantify their endogenous generation, respectively. The results indicated that [PDI(Br)_6_] NPs^•−^ increased the fluorescence of cells by 1.86‐fold for H_2_O_2_ and 1.67‐fold for •OH compared to the PBS group. Predictably, when exposed the treated cells to an 808 nm laser, the significantly enhanced fluorescence by 3.24‐fold for H_2_O_2_ and 4.75‐fold for •OH compared to the PBS group. All the results provided strong evidence for the CDT and Type‐I PDT properties of PDI(Br)_6_ NPs in tumor cells (Figure 4c; Figures S55,S56, Supporting Information). Furthermore, the therapeutic efficacy of PDI(Br)_6_ NPs toward 4T1 cells with or without laser was confirmed visually by live/dead cell costaining and flow cytometry examination (Figure 4e; Figure S57, Supporting Information). To confirm the burst release of ROS from the CDT/PDT inducing strong ICD effect, we examined the damage‐associated molecular patterns (DAMPs) on the cellular level, including monitoring ATP secretion, CRT expression, and HMGB1 release, after the treatment of PDI(Br)_6_ NPs with laser irradiation (Figure 4f; Figures S58–S60, Supporting Information). PDI(Br)_6_ NPs incubation with 4T1 cells with and without laser irradiation showed ATP release with 2.37‐fold and 4.46‐fold enhancement compared to the PBS group, respectively. The CRT exposure and HMGB1 release in 4T1 cells also showed the same tendency, indicating the PDI(Br)_6_ NPs with laser irradiation has the potential to release DAMPs for recruitment of antigen‐presenting cells and prime antitumor immune response.

### 2.4 Activable Bioimaging of PDI(Br)_6_ NPs In Vivo

**Figure 5:**
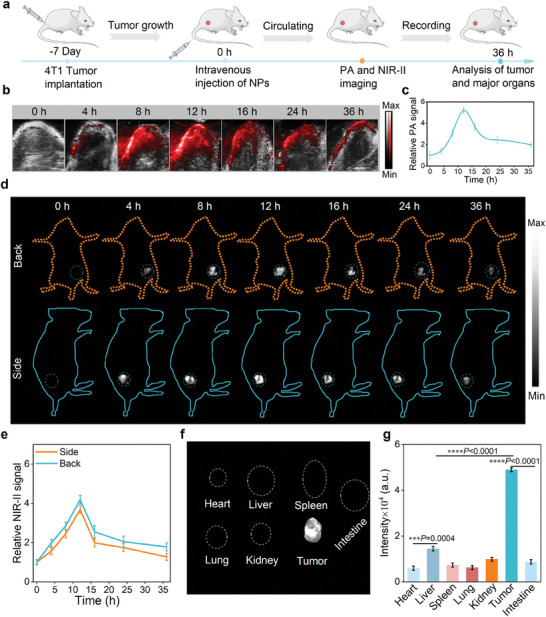
In vivo PAI and NIR‐II FLI of PDI(Br)_6_ NPs on 4T1 bearing mice. a) Experimental outline recording the PAI and NIR‐II FLI steps and procedures in 4T1 tumor‐bearing mice. Representative tumor‐specific b,c) PAI and d,e) NIR‐II FLI and their corresponding intensities of 4T1 tumor‐bearing mice at different time points after intravenous injection of PDI(Br)_6_ NPs, respectively. Data are presented as mean ± SD (*n* = 3). f,g) The NIR‐II FLI and the corresponding fluorescence intensities of the major organs and tumors from mice after 36 h post‐injection of PDI(Br)_6_ NPs. Data are presented as mean ± SD (*n *= 3).

To determine the optimal time window for photoimmunotherapy, PDI(Br)_6_ NPs were intravenously injected into 4T1 tumor‐bearing mice, and the tumor accumulation and the [PDI(Br)_6_] NPs^•−^ production were monitored by PAI and NIR‐II FLI (**Figure** **5**a). We initially explored the tumor‐specific activation capability of the PDI NPs in 4T1 tumor‐bearing mice through PA imaging under an 808 nm laser. Following the intravenous injection of PDI(Br)_6_ NPs into the 4T1 tumor‐bearing mice, time‐dependent PA images were collected (Figure 5b,c). PA intensities gradually increased over time and reached maximum signals at 12 h post‐injection, indicating the [PDI(Br)_6_] NPs accumulated tumor sites and were specifically activated into the corresponding PDI SORs.

We then investigated the activatable NIR‐II FLI of the PDI NPs by intravenously injecting of PDI(Br)_0/4/6_ NPs into 4T1 tumor‐bearing mice, respectively and time‐dependent whole‐body NIR‐II signals were collected. The NIR‐II fluorescence of tumors with PDI(Br)_4_ NPs and PDI(Br)_6_ NPs exhibited a gradual increase in tumor, and the signals reached to the peak at 12 h post‐injection. At 36 h post‐injection, we recorded the ex vivo NIR‐II FLI of the mice dissected tumor and major organs (Figure 5d–g; Figures S61,S62, Supporting Information). As proof of concept, we found that only the tumor had been lit by the PDI(Br)_4_ NPs and PDI(Br)_6_ NPs, whereas the main organs (heart, liver, spleen, lung, kidneys and intestines) showed barely visible signals. In sharp contrast, after intravenous injection of PDI(Br)_0_ NPs, no NIR‐II fluorescence signal was obtained in 4T1 tumor‐bearing mice due to the *π–π* stacking‐induced quenching of fluorescence signal (Figure S63, Supporting Information). To further explore the tumor specificity of PDI(Br)_4/6_ NPs, after intravenous injection of the PDI(Br)_4/6_ NPs into the healthy mice, no NIR‐II optical signals were detected in the healthy mice and the main organs (Figures S64,S65, Supporting Information). Conclusively, all the results validated that the PDI(Br)_4/6_ NPs enabled activatable tumor PAI/NIR‐II FLI for guiding the chemo/photodynamic combination therapy.

### 2.5 Chemo/Photodynamic Combination Therapy of PDI(Br)_6_ NPs

**Figure 6:**
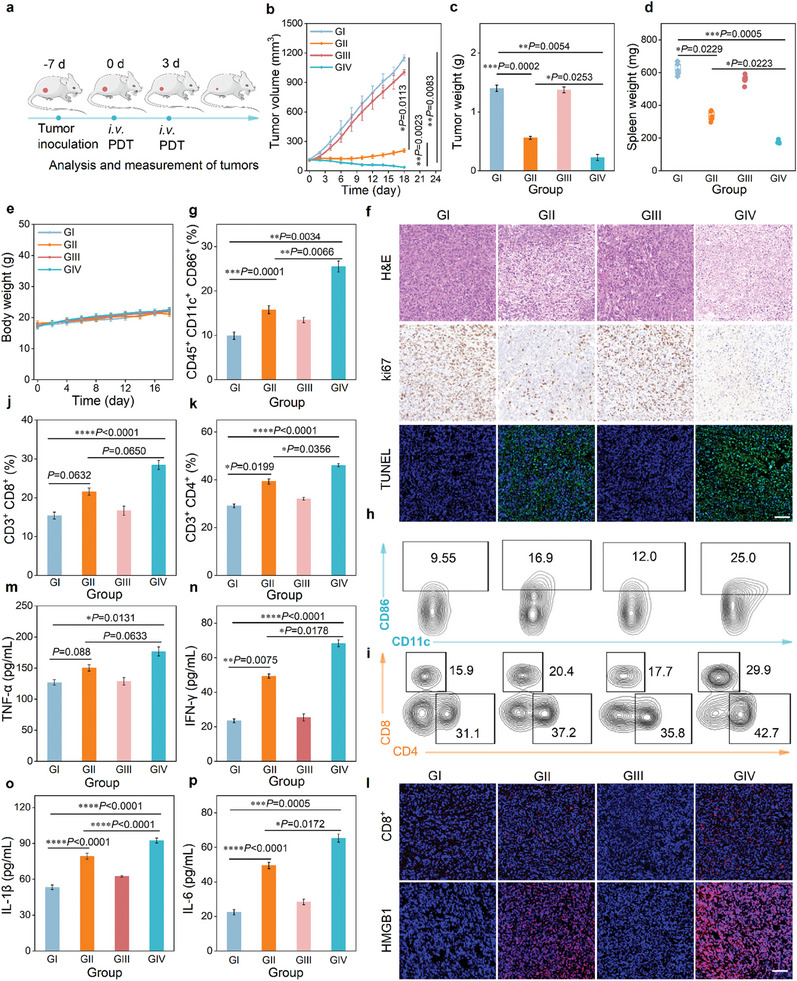
Evaluation of the in vivo therapeutic efficacy of PDI(Br)_6_ NPs. a) Experimental timeline of the treatments and procedures for evaluating the therapeutic outcomes in 4T1 tumor‐bearing mice. b) Tumor volume changes of the mice in each group during the treatment period. c) Tumor weight, d) spleen size, and e) body weight changes of mice in each group during the treatment period. Data are presented as mean ± SD (*n* = 5 mice). f) Representative H&E, Ki67, TUNEL staining of tumor sections harvested from the mice receiving various treatments. Scale bars: 100 µm. g,h) Representative flow cytometry analysis and quantitative data of matured DCs (CD45^+^ CD11c^+^ CD86^+^) in lymph nodes after various treatments. i–k) Representative flow cytometry analysis and quantitative data of the percentages of T cells (CD3^+^CD4^+^ and CD3^+^CD8^+^) were collected after various treatments. Data are presented as mean ± SD (*n* = 3 mice). l) Representative CD8^+^, HMGB1 staining of tumor sections harvested from the mice receiving different treatments. Scale bars: 100 µm. Quantification of systemic cytokines within the mice serum. m) serum TNF‐𝛼 levels, n) serum IFN‐𝛾 levels, o) serum IL‐1β levels, p) serum IL‐6 levels were analyzed by enzyme‐linked immunosorbent assay (ELISA). Data are presented as mean ± SD (*n* = 3 mice). GI: PBS, GII: PDI(Br)_6_ NPs, GIII: PBS + laser, GIV: PDI(Br)_6_ NPs + laser. Data are presented as means ± s.d. Statistical significance was calculated via One‐way ANOVA with Tukey post‐hoc test. *P < 0.05, **P < 0.01, ***P < 0.001 and ****P < 0.0001.

Encouraged by the performance of synergetic therapeutic efficacy, high tumor accumulation, and specific activation of PDI(Br)_6_ NPs, the antitumor performance in *vivo* was investigated against 4T1 tumor‐bearing mice (**Figure** **6**a). The phototherapy was conducted followed by 808 nm laser irradiation at 12 h post‐injection of PDI(Br)_6_ NPs and PBS as control. The tumor volumes and body weights of the mice were monitored after various treatments. Compared to PBS or PBS + laser treatment groups, the PDI(Br)_6_ NPs treatment group delayed tumor proliferation effects, suggesting that [PDI(Br)_6_] NPs^•−^ were specifically produced in tumors with chemodynamic ROS generation. By sharp contrast, the group treated with PDI(Br)_6_ NPs + laser displayed significant tumor suppression, indicating Type‐I PDT and CDT combination therapy could effectively inhibit tumor growth (Figure 6b; Figure S66, Supporting Information). A similar tendency was also observed in the weight of tumors after treatment (Figure 6c). In addition, by comparing the PBS or PBS+laser treatment groups, it was found that spleen size was significantly downregulated in both PDI(Br)_6_ NPs and PDI(Br)_6_ NPs + laser groups (Figure 6d). The body weights of all treated mice showed no significant changes throughout the treatment process (Figure 6e). Furthermore, major organs such as the heart, liver, spleen, lung, and kidney showed no alterations post‐treatment (Figure S67, Supporting Information). The basic blood parameters remained within a reasonable range post‐treatment (Figure S68, Supporting Information). These findings indicated that the tumor‐specific activated [PDI(Br)_6_] NPs^•−^ owning favorable biocompatibility and biosafety in the treatment. After treatment, we conducted hematoxylin and eosin (H&E) staining, TUNEL staining, and Ki67 staining analyses to verify the in vivo phototherapeutic mechanism of PDI(Br)_6_ NPs (Figure 6f). The H&E assay exhibited the extensive destruction of tumor tissues in the therapeutic groups of PDI(Br)_6_ NPs and PDI(Br)_6_ NPs + laser. In contrast, the tumor cells remained a vibrant and dense arrangement in the control groups. The severe apoptosis of PDI(Br)_6_ NPs and PDI(Br)_6_ NPs + laser groups was proved by TUNEL staining. Meanwhile, the inhibited vessel formation and suppressed cell proliferation of the tumor region were further confirmed according to the Ki67 staining. These outcomes solidly manifested that the collaborative Type‐I PDT and CDT could afford effective therapeutic efficiency.

Mature dendritic cells (DCs) are essential antigen‐presenting cells in antitumor immune responses, playing a critical role in activating CD8^+^ cytotoxic T lymphocytes (CTLs) to effectively suppress metastatic tumors. As we mentioned that the burst release of ROS by Type‐I PDT and CDT combination therapy could elicit ICD and prime antitumor immune responses, thus, we first examined the maturation of dendritic cells (DCs) in the treated mice. The relative content of mature DCs (CD45^+^CD11c^+^CD86^+^) was significantly high at 25% in the PDI(Br)_6_ NPs+laser group compared to 9.55% in the PBS group, 16.9% in the PDI(Br)_6_ NPs group, and 12% in the PBS + laser group (Figure 6g,h), suggesting the ROS‐triggered ICD effect could enhance DC maturation. CTLs (CD3^+^CD8^+^ T cells) and helper T cells (CD3^+^CD4^+^ T cells) are essential to against cancer cells, triggering an adaptive immune response. As a result, PDI(Br)_6_ NPs+laser treated tumor showed an increase in recruitment of CD3^+^CD4^+^ T cells into the tumor by 29.9%, which was 1.88, 1.68, and 1.46‐fold higher than the PBS (15.9%), PBS + laser (17.7%), and PDI(Br)_6_ NPs (20.4%) groups (Figure 6i–k). Similar results showed the recruitment of CD3^+^CD8^+^ T cells into the tumors. Furthermore, compared with PBS and PBS+laser, PDI(Br)_6_ NPs and PDI(Br)_6_ NPs+laser showed high levels of HMGB1 expression and infiltration of CD8^+^ T cells, presenting solid evidence for the PDI(Br)_6_ NPs‐triggered antitumor immune response (Figure 6l). We then assessed systemic immune responses by measuring serum levels of pro‐inflammatory cytokines including interleukin 6 (IL‐6), interleukin 1β (IL‐1β), tumor necrosis factor‐alpha (TNF‐𝛼), and interferon‐gamma (IFN‐𝛾). The ELISA assay showed a significant increase in IL‐6 and IL‐1β levels in the serum of mice treated with PDI(Br)_6_ NPs and PDI(Br)_6_ NPs + laser compared to control groups (PBS, PBS + laser), indicating a strong antitumor immune response in the experimental groups. Additionally, TNF‐𝛼 and IFN‐𝛾 levels were notably elevated after treatment with PDI(Br)_6_ NPs or PDI(Br)_6_ NPs+laser on mice (Figure 6m–p). All the results suggested that the tumor‐specific activated [PDI(Br)_6_] NPs^•−^ mediated tumor chemo/photodynamic combination therapy followed with initiating antitumor immune response and low side‐effect.

## 3 Conclusion

In summary, we obtain the four kinds of PDI(Br)_0/2/4/6_ and prepare the corresponding water‐soluble PDI(Br)_0/2/4/6_ NPs via nanoprecipitation. ESR spectroscopy and DFT‐calculated results have confirmed their open‐shell ground states, and comparative studies have further demonstrated that variations in the number of Br substituents on the PDI skeleton significantly influence the optical and ROS properties of the PDI SORs. Quantum chemical calculations and fs‐TA spectra are further performed to demonstrate an increase of Br atoms on the PDI skeleton effectively reduces intermolecular *π–π* stacking energy and improves the electron transfer rate, enhancing excited‐state charge transfer characteristics. [PDI(Br)_6_] NPs^•−^ are specifically activated in hypoxic tumors exhibiting high stability, strong PA and NIR‐II fluorescence signals. The generated [PDI(Br)_6_] NPs^•−^ show chemodynamic and photodynamic properties for eliciting a strong tumor ICD effect and exhibiting systematical safety. This study offers tumor‐specific activatable PDI SORs for “turn on” tumor theranostics, and provides new insights for developing new SORs for precision medicine.

## Conflict of Interest

The authors declare no conflict of interest.

## Supporting information



Supporting Information

## Data Availability

The data that support the findings of this study are available from the corresponding author upon reasonable request.

## References

[1] D. O. Lopez‐Cantu , R. B. González‐González , A. Sharma , M. Bilal , R. Parra‐Saldívar , H. M. Iqbal , Coord. Chem. Rev. 2022, 469, 214685.

[2] D. Chuan , H. Hou , Y. Wang , M. Mu , J. Li , Y. Ren , N. Zhao , B. Han , H. Chen , G. Guo , J. Mater. Sci. Technol. 2023, 152, 159.

[3] D. Jiang , D. Ni , Z. T. Rosenkrans , P. Huang , X. Yan , W. Cai , Chem. Soc. Rev. 2019, 48, 3683. 31119258 10.1039/c8cs00718g PMC6696937

[4] T. Wang , C. Ménard‐Moyon , A. Bianco , Chem. Soc. Rev. 2022, 51, 3535. 35412536 10.1039/d1cs01064f

[5] L. T. Huang , Z. J. Chen , Z. Yang , W. Huang , BME Front 2024, 5, 0062. 39193546 10.34133/bmef.0062 PMC11347023

[6] S. He , P. Cheng , K. Pu , Nat. Biomed. Eng. 2023, 7, 281. 36941352 10.1038/s41551-023-01009-1

[7] Y. X. Hu , J. Yu , M. K. Xu , K. Pu , J. Am. Chem. Soc. 2024, 146, 12656. 38683724 10.1021/jacs.4c02070

[8] L. K. B. Tam , J. C. H. Chu , L. He , C. Yang , K. C. Han , P. C. K. Cheung , D. K. P. Ng , P. C. Lo , J. Am. Chem. Soc. 2023, 145, 7361. 36961946 10.1021/jacs.2c13732 PMC10080691

[9] J. L. Kolanowski , F. Liu , E. J. New , Chem. Soc. Rev. 2018, 47, 195. 29119192 10.1039/c7cs00528h

[10] J. S. Wu , J. Sha , C. L. Zhang , W. M. Liu , X. L. Zheng , P. F. Wang , VIEW 2020, 1, 20200090.

[11] J. D. Knoll , B. A. Albani , C. Turro , Acc. Chem. Res. 2015, 48, 2280. 26186416 10.1021/acs.accounts.5b00227 PMC4737476

[12] X. Qin , C. Wu , D. C. Niu , L. M. Qin , X. Wang , Q. G. Wang , Y. S. Li , Nat. Commun. 2021, 12, 5243. 34475406 10.1038/s41467-021-25561-z PMC8413279

[13] G. Z. Zhou , M. Li , Adv. Mater. 2022, 34, 2200871. 10.1002/adma.202200871 35429080

[14] X. Y. Kang , Y. Zhang , J. W. Song , L. Wang , W. Li , J. Qi , B. Z. Tang , Nat. Commun. 2023, 14, 5216. 37626073 10.1038/s41467-023-40996-2 PMC10457322

[15] P. L. Cen , J. N. Huang , C. T. Jin , J. Wang , Y. Wei , H. Zhang , M. Tian , Aggregate 2023, 4, e298.

[16] J. S. Huang , L. C. Su , C. Xu , X. G. Ge , R. P. Zhang , J. B. Song , K. Pu , Nat. Mater. 2023, 22, 1421. 37667071 10.1038/s41563-023-01659-1

[17] J. Huang , Y. Jiang , J. Li , S. He , J. Huang , K. Pu , Angew. Chem., Int. Ed. 2020, 59, 4415. 10.1002/anie.201911859 31876017

[18] X. Wang , S. He , P. Cheng , K. Pu , Adv. Mater. 2023, 35, 2206510. 10.1002/adma.202206510 36317605

[19] M. Bertolini , M. S. Wong , L. Mendive‐Tapia , M. Vendrell , Chem. Soc. Rev. 2023, 52, 5352. 37376918 10.1039/d2cs00928e PMC10424634

[20] P. Cheng , S. He , C. Zhang , J. Liu , K. Pu , Angew. Chem., Int. Ed. 2023, 135, e202301625. 10.1002/anie.202301625 37099322

[21] Y. Y. Zhao , L. Zhang , Z. X. Chen , B. Y. Zheng , M. R. Ke , X. S. Li , J. D. Huang , J. Am. Chem. Soc. 2021, 143, 13980. 34425676 10.1021/jacs.1c07479

[22] M. L. Li , T. Xiong , J. J. Du , R. S. Tian , M. Xiao , L. Y. Guo , S. R. Long , J. L. Fan , W. Sun , K. Shao , X. Z. Song , J. W. Foley , X. J. Peng , J. Am. Chem. Soc. 2019, 141, 2695. 30652866 10.1021/jacs.8b13141

[23] Y. Y. Wang , Y. C. Liu , H. Sun , D. S. Guo , Coord. Chem. Rev. 2019, 395, 46.

[24] X. Li , N. Kwon , T. Guo , Z. Liu , J. Yoon , Angew. Chem., Int. Ed. 2018, 57, 11522. 10.1002/anie.201805138 29808948

[25] L. M. Loftus , A. Li , K. L. Fillman , P. D. Martin , J. J. Kodanko , C. Turro , J. Am. Chem. Soc. 2017, 139, 18295. 29226680 10.1021/jacs.7b09937 PMC5901749

[26] C. Zhang , C. Ye , J. N. Yao , L. Z. Wu , Nat. Sci. Rev. 2024, 11, 244. 10.1093/nsr/nwae244 PMC11360185 39211835

[27] X. Q. Zhao , J. Y. Gong , P. Alam , C. Ma , Y. P. Wang , J. Guo , Z. B. Zeng , Z. K. He , H. H. Y. Sung , I. Williams , K. S. Wong , S. J. Chen , J. W. Y. Lam , Z. Zhao , B. Z. Tang , CCS Chem 2022, 4, 1912.

[28] S. F. Nelsen , J. Am. Chem. Soc. 1967, 89, 5925.

[29] M. Al Kobaisi , S. V. Bhosale , K. Latham , A. M. Raynor , S. V. Bhosale , Chem. Rev. 2016, 116, 11685. 27564253 10.1021/acs.chemrev.6b00160

[30] Z. B. Zhou , K. Yang , L. He , W. Wang , W. M. Lai , Y. H. Yang , Y. G. Dong , S. Xie , L. Yuan , Z. B. Zeng , J. Am. Chem. Soc. 2024, 146, 6763. 38416700 10.1021/jacs.3c13270

[31] L. Feng , Y. Y. Tuo , Z. P. Wu , W. J. Zhang , C. B. Li , B. Yang , L. X. Liu , J. Y. Gong , G. Y. Jiang , W. Hu , B. Z. Tang , L. M. Wu , J. G. Wang , J. Am. Chem. Soc. 2024, 146, 32582. 39534977 10.1021/jacs.4c11549

[32] N. Moriyama , J. Abe , J. Am. Chem. Soc. 2023, 145, 3318. 36749150 10.1021/jacs.2c13331

[33] D. Schmidt , D. Bialas , F. Wrthner , Angew. Chem., Int. Ed. 2015, 54, 3611. 10.1002/anie.201408067 25393879

[34] T. Y. Jiao , K. Cai , J. N. Nelson , Y. Jiao , Y. Y. Qiu , G. C. Wu , J. W. Zhou , C. Y. Cheng , D. K. Shen , Y. M. Feng , Z. C. Liu , M. R. Wasielewski , J. F. Stoddart , H. Li , J. Am. Chem. Soc. 2019, 141, 16915. 31533428 10.1021/jacs.9b08926

[35] T. He , M. Stolte , F. Wurthner , Adv. Mater. 2013, 25, 6951. 24105872 10.1002/adma.201303392

[36] S. Guha , S. Saha , J. Am. Chem. Soc. 2010, 132, 17674. 21114330 10.1021/ja107382x

[37] Z. Mi , P. Yang , R. Wang , J. Unruangsri , W. Yang , C. Wang , J. Guo , J. Am. Chem. Soc. 2019, 141, 14433. 31426635 10.1021/jacs.9b07695

[38] B. Tang , W. L. Li , Y. Chang , B. Yuan , Y. Wu , M. T. Zhang , J. F. Xu , J. Li , X. Zhang , Angew. Chem., Int. Ed. 2019, 58, 15526. 10.1002/anie.201910257 31478324

[39] I. Ghosh , T. Ghosh , J. I. Bardagi , B. König , Science 2014, 346, 725. 25378618 10.1126/science.1258232

[40] P. Ravat , T. Solomek , D. Haussinger , O. Blacque , M. Juricek , J. Am. Chem. Soc. 2018, 140, 10839. 30067898 10.1021/jacs.8b05465 PMC6120736

[41] T. J. Whittemore , A. Millet , H. J. Sayre , B. S. Dolinar , E. G. White , K. R. Dunbar , C. Turro , J. Am. Chem. Soc. 2018, 140, 5161. 29617115 10.1021/jacs.8b00599

[42] G. E. Rudebusch , J. L. Zafra , K. Jorner , K. Fukuda , J. L. Marshall , I. Arrechea‐Marcos , G. L. Espejo , R. P. Ortiz , C. J. Gomez‐Garcia , L. N. Zakharov , M. Nakano , H. Ottosson , J. Casado , M. M. Haley , Nat. Chem. 2016, 8, 753. 27442280 10.1038/nchem.2518

[43] H. Gao , X. Zhi , F. Wu , Y. Zhao , F. Cai , P. Li , Z. Shen , Angew. Chem., Int. Ed. 2023, 62, e202309208. 10.1002/anie.202309208 37590036

[44] R. Englman , J. Jortner , Mol. Phys. 1970, 18, 145.

[45] X. Cui , G. Lu , S. Dong , S. Li , Y. Xiao , J. Zhang , Y. Liu , X. Meng , F. Li , C. S. Lee , Mater. Horiz. 2021, 8, 571. 34821273 10.1039/d0mh01312a

[46] B. B. Zhang , R. J. Zheng , Y. T. Liu , X. Lou , W. Zhang , Z. J. Cui , Y. W. Huang , T. Wang , Adv. Sci. 2022, 10, 2204498. 10.1002/advs.202204498 PMC9875625 36373677

[47] X. Lou , H. Wang , Y. Liu , Y. W. Huang , Z. H. Liu , W. Zhang , T. Wang , Angew. Chem., Int. Ed. 2023, 62, e202214586. 10.1002/anie.202214586 36597125

[48] J. Zhang , W. Ma , H. F. Luo , K. X. Zhang , J. Q. Lv , L. Z. Jiang , Y. L. Huang , J. B. Song , Z. Yang , W. Huang , Adv. Healthcare Mater. 2024, 13, 2303175. 10.1002/adhm.202303175 37985358

[49] Y. Kumar , S. Kumar , K. Mandal , P. Mukhopadhyay , Angew. Chem., Int. Ed. 2018, 57, 16318. 10.1002/anie.201807836 30260056

[50] H. C. Mao , G. J. Pazera , R. M. Young , M. D. Krzyaniak , M. R. Wasielewski , J. Am. Chem. Soc. 2023, 145, 6585. 36913602 10.1021/jacs.3c01243

[51] Y. L. Wu , N. E. Horwitz , K. S. Chen , D. A. Gomez‐Gualdron , N. S. Luu , L. Ma , T. C. Wang , M. C. Hersam , J. T. Hupp , O. K. Farha , R. Q. Snurr , M. R. Wasielewski , Nat. Chem. 2016, 9, 466. 28430197 10.1038/nchem.2689

[52] L. Zeng , L. Huang , Z. Huang , T. Mani , K. Huang , C. Y. Duan , G. Han , Nat. Commun. 2024, 15, 7270. 39179545 10.1038/s41467-024-50795-y PMC11344023

[53] Y. Jiao , K. Liu , G. T. Wang , Y. P. Wang , X. Zhang , Chem. Sci. 2015, 6, 3975. 29218167 10.1039/c5sc01167a PMC5707502

[54] Y. F. Wu , S. W. Ying , L. Y. Su , J. J. Du , L. Zhang , B. W. Chen , H. R. Tian , H. Xu , M. L. Zhang , X. Yan , J. Am. Chem. Soc. 2022, 144, 10736. 35671378 10.1021/jacs.2c00794

[55] C. J. Zeman , S. J. Kim , F. Zhang , K. S. Schanze , J. Am. Chem. Soc. 2020, 142, 2204. 31927964 10.1021/jacs.9b13027

[56] K. Zhou , L. L. Du , R. Ding , L. T. Xu , S. Shi , S. Y. Wang , Z. Y. Wang , G. Q. Zhang , G. He , Z. Zhao , B. Z. Tang , Nat Commun. 2024, 15, 10551. 39632877 10.1038/s41467-024-55060-w PMC11618361

[57] Z. J. Zhao , F. S. Niu , P. J. Li , H. Q. Wang , Z. H. Zhang , G. J. Meyer , K. Hu , J. Am. Chem. Soc. 2022, 144, 7043. 35271254 10.1021/jacs.2c00422

[58] X. M. Hu , Z. T. Fang , F. W. Sun , C. J. Zhu , M. Y. Jia , X. F. Miao , L. T. Huang , W. B. Hu , Q. L. Fan , Z. Yang , W. Huang , Angew. Chem., Int. Ed. 2024, 63, e202401036. 10.1002/anie.202401036 38362791

[59] X. Miao , W. B. Hu , T. He , H. Tao , Q. Wang , R. Chen , L. Jin , H. Zhao , X. Lu , Q. L. Fan , W. Huang , Chem. Sci. 2019, 10, 3096. 30996892 10.1039/c8sc04840a PMC6429462

[60] H. Wang , K. F. Xue , Y. C. Yang , H. Hu , F. J. Xu , X. Zhang , J. Am. Chem. Soc. 2022, 144, 2360. 35051337 10.1021/jacs.1c13067

[61] L. Zhao , Y. Liu , R. Xing , X. Yan , Angew. Chem., Int. Ed. 2020, 59, 3793. 10.1002/anie.201909825 31571353

[62] X. J. Zhao , R. J. Zheng , B. B. Zhang , Y. Zhao , W. L. Xue , Y. F. Fang , Y. W. Huang , M. Z. Yin , Angew. Chem., Int. Ed. 2024, 136, e202318799. 10.1002/anie.202318799 38230819

[63] C. Ji , Q. Gao , X. Dong , W. Yin , Z. Gu , Z. Gan , Y. Zhao , M. Z. Yin , Angew. Chem., Int. Ed. 2018, 57, 11384. 10.1002/anie.201807602 30003656

[64] J. Zhao , W. Wu , J. Sun , S. Guo , Chem. Soc. Rev. 2013, 42, 5323. 23450221 10.1039/c3cs35531d

[65] S. M. Dyar , E. A. Margulies , N. E. Horwitz , K. E. Brown , M. D. Krzyaniak , M. R. Wasielewski , J. Phys. Chem. B 2015, 119, 13560. 26010882 10.1021/acs.jpcb.5b02378

[66] R. Lincoln , L. Kohler , S. M. A. Monro , H. M. Yin , M. Stephenson , R. Zong , A. Chouai , C. L. Dorsey , R. A. Hennigar , R. S. M. Thummel , J. Am. Chem. Soc. 2013, 135, 17161. 24127659 10.1021/ja408426z

[67] K. Ishii , Y. Hirose , H. Fujitsuka , O. Ito , N. Kobayashi , J. Am. Chem. Soc. 2001, 123, 702. 11456584 10.1021/ja002780h

[68] C. Michael T , G. Emilie M , C. Boiko , M. Tomoaki , S. Amy M , W. Michael R , J. Phys. Chem. A 2010, 114, 1741.20055506

[69] Y. Teki , S. Miyamoto , K. Iimura , M. Nakatsuji , Y. Miura , J. Am. Chem. Soc. 2000, 122, 984. 10.1021/ja001920k 11456516

