# Supplementary material for: Semiconducting Open‐Shell Radicals for Precise Tumor Activatable Phototheranostics
Source: Adv Sci (Weinh). 2025 Mar 7;12(17):2500293. doi: 10.1002/advs.202500293 (PMC12061280; doi:10.1002/advs.202500293)
Supplement: Supplementary file 1 — Supporting Information [file ADVS-12-2500293-s001.docx]

Supplementary information

**Semiconducting Open-shell Radicals for Precise Tumor Activatable Phototheranostics**

Jie Zhang,**^†^** Haifen Luo,**^†^** Wen Ma, Jingqi Lv, Bo Wang, Fengwei Sun, Weijie Chi,**^*^** Zhuting Fang,^*^ Zhen Yang^*^

Dr. J. Zhang, H. F. Luo, W. Ma, J. Q. Lv, B. Wang, F. W. Sun, Prof. Z. Yang

Strait Laboratory of Flexible Electronics (SLoFE), Fujian Key Laboratory of Flexible Electronics, Strait Institute of Flexible Electronics (Future Technologies), Fujian Normal University, Fuzhou 350117, China.

E-mail: [ifezhyang@fjnu.edu.cn](mailto:ifezhyang@fjnu.edu.cn)

Prof. Z. T. Fang

Department of Oncology and VascuarInterventonal Therapy, Clinical OncologySchool of Fujian Medical University, Fujian Cancer Hospital,Fuzhou,350014, China.

Department of imterventional Radiology, shengli Clinical Medical College of Fujian Medical University, Fuijian Provincial Hospital, Fuzhou University Affliated Provincial Hospital, Fuzhou 350001. China.

E-mail: ztfang@fjzlhospital.com

Prof. W. J. Chi

School of Chemistry and Chemical Engineering, Hainan University, Haikou 570228, China.

E-mail: [weijie_chi@hainanu.edu.cn](mailto:weijie_chi@hainanu.edu.cn)

**^†^**These authors contributed equally: J. Zhang, H. F. Luo

**Table of Contents**

1. **General information……………………..……..…………………….**6

1.1. Materials……………..……….…………..………………………….....6

1.2. Instruments..……………..…………………………...............................7

**2. Computational details****……………..…………….………....................**7

**3. General methods…………………………………….……….……**7-15

**4. Synthetic and characterization………………….............…….........**16

4.1 Synthesis of PDI(Br)_0_ (Scheme S1)…..…………………………………..............16

4.2 Synthesis of PDI(Br)_2_ (Scheme S2)…..…………………………………..............17

4.3 Synthesis of PDI(Br)_4_ (Scheme S3).…..………………………………………….18

4.4 Synthesis of PDI(Br)_6_ (Scheme S4).…..……………………………..……..…….19

**5. Supplementary figures…..……………………......…........................**20

Figure S1. ^1^H NMR spectrum of BADT………………………....................................20

Figure S2. ^1^H NMR spectrum of PDI(Br)_0_..……………………………………..……20

Figure S3. HR-MS spectrum of PDI(Br)_0_..……………………………………….…..21

Figure S4. ^1^H NMR spectrum of 2Br-BADT.………………………............................21

Figure S5. ^1^H NMR spectrum of PDI(Br)_2_..……………………………………..…....22

Figure S6. HR-MS spectrum of 2Br-BADT.……………………………………….....22

Figure S7. HR-MS spectrum of PDI(Br)_2_....………………………………………….22

Figure S8. ^1^H NMR spectrum of 4Br-BADT……………………………………........23

Figure S9. ^1^H NMR spectrum of PDI(Br)_4_..………………………………………..…23

Figure S10. HR-MS spectrum of 4Br-BADT.………………………...........................23

Figure S11. HR-MS spectrum of PDI(Br)_4_...………………………………………...24

Figure S12. ^1^H NMR spectrum of 6Br-BADT……………………………………......24

Figure S13. ^1^H NMR spectrum of PDI(Br)_6_..…………………………………….......24

Figure S14. HR-MS spectrum of 6Br-BADT.……………………………………..…25

Figure S15. HR-MS spectrum of PDI(Br)_6_....………………………...……………...25

Figure S16. Zeta potential analysis..…………….…………………............................25

Figure S17. DLS analysis….……….………….………………………….………….26

Figure S18. Stability of nano-solutions.……….………………………..………….…26

Figure S19. Absorption and fluorescence spectrum of PDI(Br)_n_ NPs………………...27

Figure S20. Fluorescence spectrum changes of PDI(Br)_4/6_ NPs with the addition of reducing agents (Na_2_S_2_O_3_, Na_2_S_2_O_4_)…….…………………………………………...27

Figure S21. The absorption spectrum of [PDI(Br)_n_] NPs^•−^ produced in the presence of different amounts of Na_2_S_2_O_4_…….………………………………………..................28

Figure S22. The absorption of [PDI(Br)_n_] NPs^•−^ were produced in the presence of different amounts of Na_2_S_2_O_3_….…………………......................................................28

Figure S23. Cyclic voltammograms of PDI(Br)_n_..………………………..………......29

Figure S24. Calculated frontier molecular orbitals and energy levels of the PDI(Br)_0/2_…………………………………………………………………………….29

Figure S25. Calculated frontier molecular orbitals and energy levels of the PDI(Br)_4/6_,……………………………………………………………………………30

Figure S26. The NIR-II fluorescence spectrum of PDI(Br)_n_ NPs in the presence of different amounts of Na_2_S_2_O_4_….……………………………………….....................30

Figure S27. The NIR-II fluorescence spectrum of PDI(Br)_n_ NPs in the presence of different amounts of Na_2_S_2_O_3_….……………………….……………….....................31

Figure S28. The stability of [PDI(Br)_4_] NPs^•−^ under hypoxia conditions……….……31

Figure S29. The stability of [PDI(Br)_4_] NPs^•−^ under different pH conditions……......32

Figure S30. The stability of [PDI(Br)_6_] NPs^•−^ under different pH conditions.………...32

Figure S31. The stability of [PDI(Br)_4/6_] NPs^•−^ under FBS…………………………...33

Figure S32. Photostability test of [PDI(Br)_4/6_] NPs^•−^ …………..……………………..33

Figure S33. Photothermal stability of [PDI(Br)_4/6_] NPs^•−^………….……………….....34

Figure S34. The dihedral angles of four PDIs…………………………………………34

Figure S35. The dihedral angles of four PDIs^•−^ …………..…………………..............34

Figure S36. The SUMO orbitals and energy levels of four PDIs^•−^…………………….35

Figure S37. The changing amount of generated radical anions with increasing reductant (Na_2_S_2_O_3_ solution) …………………………………..………….................................35

Figure S38. Mechanism of DCFH，DHR123 and HPF as an indicator for ROS, superoxide radical and hydroxyl radicals…………………………………….……....35

Figure S39. Detection of ROS………………………………..………….……...….....36

Figure S40. Detection of endogenous ROS………………………………………….36

Figure S41. Detection of O_2_^•-^…………………………………………….……..…….37

Figure S42. Detection of O_2_^•-^ detection under hypoxic conditions……………….…..37

Figure S43. Detection of endogenous O_2_^•-^………………...………….……….……..38

Figure S44. Detection of •OH.………………………………..………..…………......39

Figure S45. Detection of endogenous •OH..……………………………..…………..40

Figure S46. Detection of ^1^O_2_ …………………………….……...………………..…..40

Figure S47. ESP of PDI(Br)_0/2_…………………….…...…………..……………….....41

Figure S48 ESP of [PDI(Br)_0/2_]^•−^……………….………………………..………...….41

Figure S49. Femtosecond transient absorption spectroscopy……………….………..42

Figure S50. The NIR-II fluorescence intensity of normal and tumor cells……….....42

Figure S51. The stability of PDI^•−^ by ESR spectrum.…................................................43

Figure S52. Spin density distribution of [PDI(Br)_0/2_]^•−^…………………...…………..43

Figure S53. Spin density distribution of [PDI(Br)_4/6_]^•−^…………………………….....44

Figure S54. Flow cytometry analysis of total ROS in 4T1 cells……………………..44

Figure S55. Representative fluorescence images of H_2_O_2_ in 4T1 cells…………….…44

Figure S56. Representative fluorescence images and flow cytometry analysis of •OH in 4T1 cells..…………………………………………………………………………….45

Figure S57. Live and dead cells imaging……………………………………………..45

Figure S58. Detection of ATP secreted into the medium.……………………………46

Figure S59. Images of the HMGB1 release ………………………………….……...46

Figure S60. Images of the CRT exposure………………………………………….....47

Figure S61. NIR-II FLI of PDI(Br)_4_ NPs in 4T1 tumor-bearing mice………..……...47

Figure S62. Ex vivo NIR-II FLI of major organs of PDI(Br)_4_ NPs).………….……...48

Figure S63. NIR-II FLI of PDI(Br)_0_ NPs in 4T1 tumor-bearing mice……………….48

Figure S64. NIR-II FLI of PDI(Br)_6_ NPs in healthy normal BALB/c mice.……........49

Figure S65. NIR-II FLI of PDI(Br)_4_ NPs in healthy normal BALB/c mice..………….50

Figure S66. The tumor volume changes…………………..……………….…………51

Figure S67. H&E staining analysis of major organs…………………....………...….51

Figure S68. Blood biochemistry test…………………………..…...……………….51

**6. Supplementary references.……………….…..…..………………..**52

**1. General information**

**1.1. Materials**

3,4,9,10-perylene tetracarboxylic acid dianhydride and benzyl bromide were purchased from Energy Chemical. N-methylpyrrolidone, N-Bromosuccinimide, benzyl bromide, methanol, tetrahydrofuran (THF) and N, N-dimethyl-1,3-propanediamine were purchased from Aladdin. (Shanghai, China). Concentrated sulfuric acid were purchased from Sinopharm Chemical Reagent Co., Ltd. (Shanghai, China). All other chemicals and reagents were purchased and used without any further purification. Phosphate buffered solution (PBS, pH 5.0, 6.0, 6.5, 7.4) were purchased from Aladdin reagent. (Shanghai, China). 2',7'-dichlorodihydrofluorescein diacetate (DCFH-DA), singlet oxygen sensor green (SOSG), dihydrorhodamine 123 (DHR123), hydroxyphenyl fluorescein (HPF), calcein acetoxymethyl ester (Calcein-AM), propidium iodide (PI) were purchased from Sigma-Aldrich. (St. Louis, USA). Detector dihydroethidium (DHE), Cell Counting Kit-8 (CCK-8) was purchased from GLPBIO. Calcein-AM/PI staining kit was obtained from KeyGEN BioTECH Co., Ltd. (China). Annexin V-FITC/PI staining kit was purchased from Yeasen Biotechnology (Shanghai) Co., Ltd. (China). Penicillin-Streptomycin Solution was from Beijing Solarbio Science & Technology Co., Ltd. Dulbecco’s modified eagle medium (DMEM) was from Pricella Life Science&Technology Co., Ltd. H_2_O_2_ probe was from Maokangbio, MKBio, Maokang Biotech. Fetal Bovine Serum (FBS) was form ExCell Bio, China. All the ELISA kits were obtained from Elabscience® Biotechnology Co., Ltd. (China).

**1.2. Instruments.**

^1^H NMR spectra were recorded with a Bruker ARX 600 NMR spectrometer using tetramethylsilane (TMS) as a reference at room temperature. The high-resolution mass spectra (HRMS) were collected with GCT Premier CAB 048 mass spectrometer in MALDI-TOF mode. The UV/Vis–NIR absorption spectrum were measured by a SHIMADZU UV-2600i spectrophotometer. The fluorescence spectroscopy was conducted on a FS5 spectrofluorometer. Size distribution was analyzed on a dynamic light scattering (DLS) using an Omni Nano Brook. Electronic paramagnetic resonance (EPR) measurements were performed on a JEOLJES-FA200 apparatus, with 9048 MHz microwave frequency and 0.20 mW power. Photodynamic and photothermal experiments were implemented by using 808 nm infrared semiconductor laser (CNIlaser, MDL-XD-808-2.5W). Temperature change was monitored by an FLIR E8-XT camera (FLIR System). The cell viability was detected by CCK-8 kit or MTT assay kit, and the absorbance of each sample was measured at 450 nm or 490 nm using a microplate reader (BioTek). The cellular fluorescence images were taken by inverted fluorescence microscope. The in *vivo* NIR-II fluorescence was imaged by the NIR-II fluorescence imaging instrument (NIR-30F).

**2. Computational details**

The geometrical structures of PDI derivatives were optimized using ω-B97XD functional with Def2SVP basis set. The solvent model density (SMD) solvation model was used to simulate the solvent effect in chloroform. The dimeric structures of PDI derivatives were builded according to the distributions of electrostatic potential. All calculations were carried out with Gaussian 16 A and took into account van der Waals correction.

**3. General methods**

**PDI NPs were gained**

Preparation of Nanoparticles: **PDI NPs** were synthesized through nanoprecipitation of Pluronic® F-127 and PDI in a mixture of water and THF or THF/CH_3_OH under continuous sonication. In short, PDI (1 mg), and Pluronic® F-127 (10 mg) were dispersed in 1 mL of THF or THF/CH_3_OH, which was fleetly added into the mixed liquor containing THF/CH_3_OH (1 mL) and deionized water (9 mL) with persistent sonication for 5 min. THF/CH_3_OH was then removed by decompression at 40 °C. The resulting complexes were further purified via the centrifugal filter (molecular weight cutoff: 10 kDa) to remove the unreacted Pluronic® F-127 chains and superfluous impurities.

**[PDI(Br)_4_] NPs^•－^ and [PDI(Br)_6_] NPs^•－^ were gained.**

Fresh Na_2_S_2_O_3_ compound was taken and added to aqueous solutions of PDI(Br)_4_ NPs and PDI(Br)_6_ NPs (0.3 mM, 10 mL) under sonication conditions. The mixture solutions were stirred at room temperature for 30 min and continuously monitored by UV/Vis–NIR spectroscopy. The excess Na_2_S_2_O_3_ was removed by ultrafiltration centrifugation method. The fresh PDI(Br)_4_ NPs and PDI(Br)_6_ NPs radical anions were obtained and stored in a 4 ^o^C environment for subsequent experiments.

**Storage stability.**

The concentrated aqueous solution of as-prepared PDI NPs was diluted with PBS and distilled water. At the designated time, an aliquot of the solution was taken and monitored by DLS. The PDI NPs**^•－^** were also diluted with 10% fetal bovine serum (FBS) and buffer solutions with different pH to assess their stability.

**ESR spectrum of PDI NPs**^•−^**.**

The tumor and normal cells were seeded in a ESR sample tube with a density of 2 x 10^6^ cells/mL. The culture media were DMEM with 0.20 mM PDI NPs. The ESR sample tubes containing the tumor cells were sealed in hypoxic conditions, then placed in an incubator (37°C) for 24 h. The ESR sample tubes with the normal cells were placed in normoxic conditions, then placed in an incubator (37°C) for 24 h. The samples in ESR sample tubes were characterized for ESR signals under room temperature.

**Total ROS Detection by DCFH.**

Dichlorodihydrofluorescein (DCFH) fluorescence intensity was applied to detect reactive oxygen species. Typically, 30 μL DCFH (concentration: 30 μM) was mixed with the [PDI(Br)_4_] NPs^•－^, [PDI(Br)_6_] NPs^•－^ solution. Control group were prepared by adding 3 mL PBS in 30 μL DCFH. After that, the mixtures were exposed to 808 nm irradiation (0.30 W cm^-2^), and the FL intensities at ~530 nm were recorded in a FL spectrofluorometer (Excitation wavelength: 488 nm).

**Detection of ^1^O_2_ Generation by SOSG.**

^1^O_2_ generation in solution were monitored with SOSG by its FL intensity. Briefly, SOSG in DMSO (working concentration: 30 μM) was mixed with the [PDI(Br)_4_] NPs^•－^, [PDI(Br)_6_] NPs^•－^ solution and irradiated by 808 nm laser (0.30 W cm^-2^). Control group were prepared by adding 3 mL PBS in 20 μL SOSG. Then, the FL intensity at ~530 nm was recorded in a FL spectrofluorometer (Excitation wavelength: 500 nm).

**Detection of** **O_2_^•−^ Generation by DHR123**

For the detection of O_2_^•−^ in solution, the compound dihydrorhodamine 123 (DHR123) was used as an indicator. When O_2_^•−^ is produced in the system, the DHR123 is oxidized and emits a strong fluorescence. Typically, DHR123 in DMSO (concentration: 30 μM) was mixed with the [PDI(Br)_4_] NPs^•－^, [PDI(Br)_6_] NPs^•－^ solution. The control group was made by combining 3 mL PBS with 20 mL DHR123. The mixture was then placed in a cuvette and irradiated by 808 nm laser (0.30 W cm^-2^). The fluorescence change of the sample at 525 nm was recorded by the FL spectrofluorometer (excitation wavelength: 488 nm).

**Detection of •OH Generation by HPF**

Compound hydroxyphenyl fluorescein (HPF) was used as indicator for detection of •OH in solution. When •OH is generated in the system, the HPF will be oxidized and emit strong fluorescence centered at ~ 515 nm. Typically, HPF in DMF (concentration: 30 μM) was mixed with the [PDI(Br)_4_] NPs^•－^, [PDI(Br)_6_] NPs^•－^ mixed H_2_O_2_ solution. Control group were prepared by adding 3 mL PBS in 20 μL HPF. The mixture was then placed in a cuvette and irradiated by 808 nm laser (0.30 W cm^-2^). The fluorescence change of sample was recorded by the FL spectrofluorometer (Excitation wavelength: 490 nm).

**Electron Spin Resonance (ESR) Experiment for O_2_^•−^**

ESR experiment was used to identify the type of ROS using 5-tert-butoxycarbonyl-5-methyl-1-pyrroline-N-oxide (BMPO) as the radical indicator. [PDI(Br)_4_] and [PDI(Br)_6_] NPs^•−^ containing BMPO (DMSO: H_2_O = 1: 9, v/v) before and after laser for 10 min. [BMPO] = 0.15 M.

**Electron Spin Resonance (ESR) Experiment for •OH**

ESR experiment was used to identify the type of ROS using 5,5-dimethyl-1-pyrroline-N-oxide (DMPO) as the radical indicator. [PDI(Br)_4_] and [PDI(Br)_6_] NPs^•−^ containing DMPO (DMSO: H_2_O = 1: 9, v/v) before and after laser for 10 min. [DMPO] = 0.15 M. H_2_O_2_ was added to all test solutions.

**Femtosecond Transient Absorption Spectroscopy**

The femtosecond transient absorption setup is based on a regenerative amplified Ti:sapphire laser system from Coherent (750 nm, 35 fs, 6 mJ/pulse, and 1 kHz repetition rate), nonlinear frequency mixing techniques and the Helios spectrometer (Ultrafast Systems LLC). Briefly, the 750 nm output pulse from the regenerative amplifier was split in two parts with a 50% beam splitter. The transmitted part was used to pump a TOPAS Optical Parametric Amplifier (OPA) which generates a wavelength-tunable laser pulse from 250 nm to 2.5 μm as pump beam. The reflected 750 nm beam was split again into two parts. One part with less than 10% was attenuated with a neutral density filter and focused into a 2 mm thick sapphire window to generate a white light continuum (WLC) from 600 nm to 1000 nm used for probe beam. The probe beam was focused with an Al parabolic reflector onto the sample. After the sample, the probe beam was collimated and then focused into a fiber-coupled spectrometer with CMOS sensors and detected at a frequency of 1 KHz. The intensity of the pump pulse used in the experiment was controlled by a variable neutral-density filter wheel. The delay between the pump and probe pulses was controlled by a motorized delay stage. The pump pulses were chopped by a synchronized chopper at 500 Hz and the absorbance change was calculated with two adjacent probe pulses (pump-blocked and pump-unblocked). All experiments were performed at room temperature

**Intracellular ROS measurement under hypoxic conditions.**

Organisms and growth conditions. 4T1 cells was maintained in dulbecco's modified eagle medium (DMEM) supplemented with 10% fetal bovine serum (FBS, v/v), penicillin (50 units·mL^-1^), and streptomycin (50 μg·mL^-1^) at 37℃ in a humidified 5% CO_2_ atmosphere. The intracellular ROS was examined by using 2,7-dichlorofluorescein diacetate (DCFH-DA) as a fluorescence probe. 4T1 cells were incubated with PDI(Br)_6_ NPs for 12 h followed by incubation with 10 μM DCFH-DA for 30 min under hypoxic conditions. After then washed with PBS for two times, cells were irradiated with the 808 nm light (0.3 W·cm^-2^ for 10 min). Then, the fluorescence was immediately observed with the excitation wavelength of 488 nm, and emission collection wavelength was 510 nm to 550 nm. For O_2_^•−^ detection, 4T1 cells were incubated with PDI(Br)_6_ NPs for 12 h, followed by incubation with 10 μM DHE for another 30 min. After being washed by PBS for three times, cells were irradiated with 808 nm light (0.3 W·cm^-2^ for 10 min). Then the cells were immediately observed with the excitation wavelength of 511-551 nm, and emission collection wavelength from 573-613 nm. Hydrogen peroxide and hydroxyl radical were determined with H_2_O_2_ probe and HPF as indictors, respectively

**In vitro cytotoxicity analysis.**

The cytotoxicity of different formulations was determined by cell counting kit-8 (CCK-8) assays in 4T1 or L929 cells. Briefly, cells were seeded in 96-well plates (1×10^4^ per well) and cultured for 24 h. 200 μL of culture medium containing different concentrations of PDI(Br)_6_ NPs was added and cultured for 8 h, some of them were treated with 808 nm laser (0.3 W cm^-2^) for 5 min, followed by incubation for another 20 h. CCK-8 was then added to each well and incubated for another time. After the incubation, the results were obtained at 450 nm.

**Live/Dead cell co-staining**.

For live/dead cells co-staining assay, 1.5×10^5^ 4T1 cells were seeded and cultured in 6-well plates and incubated overnight at 37℃ in a humidified 5% CO_2_ atmosphere for cell adherence and growth. Then the cells were exposed to the different following treatments: PBS, PBS+laser, PDI(Br)_6_ NPs, PDI(Br)_6_ NPs+laser using an ice-bath to inhibit the temperature elevation of cells during light treatment, light power density was 0.3 W/cm^2^. After 4T1 cells were subjected to above different treatments, the cells were further stained with Calcein-AM/PI Double Stain Kit according to the instruction manual. The excitation wavelength was 488 nm, and emission wavelength was collected from 505 to 545 nm for green channel and from 600 to 700 nm for red channel.

**CRT detection**

After cells were completely adherent to the wall in 20 mm culture dish, PDI(Br)_6_ NPs solution was putted in and continued to culture for 12 h. Subsequently, the solution was exposed to 808 nm (0.3 W cm^-2^) laser irradiation. Then, Different groups of cells were washed with PBS and were fixed by methanol for 5 min. Next, primary antibody was added in each group at 4℃ overnight and then was removed in the next day. Finally, the cells were incubated with the secondary antibody (1:500 dilution) of goat antirabbit IgG-Alexa 594 in each group for 2 h at 37℃ and then Hoechst for 15 min after cleaning with PBS. Confocal laser microscopy was used to observe and take pictures.

**HMGB1 detection.**

After cells were completely adherent to the wall in 20 mm culture dish, PDI(Br)_6_ NPs solution was putted in and continued to culture for 8 h. Subsequently, the solution was exposed to 808 nm (0.3 W cm^-2^) laser irradiation. Then, Different groups of cells were washed with PBS and were fixed by methanol (5 min). Next, PBS containing 1% Triton X-100 was injected into culture dish a bated with cells (5 min). Subsequently, primary antibody was added in each group at 4°C overnight and then removed in the next day. Finally, the cells were incubated with goat anti-rabbit IgG-Alexa 488 secondary antibody (1:250 dilution) in each group for 2 h (37°C) and then Hoechst for 15 min after cleaning with PBS. Confocal laser microscopy was used to observe and take pictures.

**The release of ATP**

After cells were completely adherent to the wall in 20 mm culture dish, PDI(Br)_6_ NPs solution was putted in and continued to culture for 12 h. Subsequently, the solution was exposed to 808 nm (0.3 W cm^-2^) laser irradiation. Then the culture medium was collected to the following test. After 100 μL ATP detection working solution and 20 μL medium was added into each well, and the fluorescence intensity was detected by Microplate Spectrophotometer. The standard curve of ATP was constructed with fluorescence intensity as longitudinal axis and ATP concentration as lateral axis. The fluorescence values of each sample were taken into the standard curve to calculate ATP concentration.

**In vivo subcutaneous tumor models**

All animal experiments have been approved by the Animal Management and Ethics Committee of Fujian Normal University (IACUC-20230045). The 5-week-old BALB/c mice were raised in SPF animal room. A suspension of 2×10^6^ 4T1 cells in PBS solution was inoculated subcutaneously in the right lower limb of mice. 4T1 tumor-bearing model were randomized into control group and treatment group (10 mice in each group). After the tumor grew to 100 mm^3^, the control group was treated with physiological saline, and the treatment group was treated with 2 mg /kg^-1^ PDI(Br)_6_ NPs every 2 days for 2 times. The change of tumor volume and weight of mice were recorded every 2 days. After the treatment, the mice were euthanized, and the eyeball blood, main organs and tumor tissues were collected for analyses, and then the eyeball blood was collected to test the cytokines and other indicators while the tissues were fixed for subsequent experiments.

**In vivo photoacoustic imaging**

All animal experiments have been approved by the Animal Management and Ethics Committee of Fujian Normal University (IACUC-20230045). 4T1 cells were transplanted in the subcutaneous of BALB/c mice at the density of 2 × 10^5^ cells per mouse. Once the tumor volume reached 100-120 mm^3^, the mice were intravenously injected with PDI(Br)_6_ NPs. The in vivo photoacoustic imaging was examined.

**In vivo NIR-II fluorescence imaging.**

All animal experiments have been approved by the Animal Management and Ethics Committee of Fujian Normal University (IACUC-20230045). 4T1 cells were transplanted in the subcutaneous of BALB/c mice at the density of 2 × 10^6^ cells per mouse. Tumors were grown for 7 days before use. To evaluate the tumor concentration of PDI(Br)_0_ NPs, PDI(Br)_4_ NPs, PDI(Br)_6_ NPs respectively, the tumor NIR-II fluorescence imaging was recorded with intravenous injection of PDI(Br)_0_ NPs, PDI(Br)_4_ NPs, PDI(Br)_6_ NPs, and the following images were collected at different post-injection times. At 36 hours post-injection, the mice were euthanized and NIR-II fluorescence images of the normal tissues and tumors were collected using NIR-30F.

**Cytokines detection*.***

After the treatment, mice blood was collected after anesthesia and placed in tubes containing anticoagulant heparin sodium and serum was centrifuged for analysis. ELISA kits were used to detect based on the standard protocols.

**Flow cytometric analysis the Ex vivo immune response.**

In order to assess the level of DC maturation in vivo, mice were sacrificed after various treatments and tumor-draining lymph nodes were collected. After single-cell suspension, the collected cells from draining lymph nodes were stained with FITC anti-mouse CD86, APC anti-mouse CD80 and PE anti-mouse CD11C based on vendor’s protocols. Then the level of matured DCs (gated as CD11c^+^CD45^+^CD86^+^ cells) and T cells (CD3^+^ CD4^+^, CD3^+^ CD8^+^) were analyzed by flow cytometry. In order to further evaluate the immune response, mice treated with various therapies were sacrificed, and subcutaneous tumor and spleens were collected and then fabricated into single cell suspension based on standard protocols. In other words, tumors were cut into tiny pieces and then digested with mixture containing collagenase type IV (200 U mL^-1^) and DNase I (40 U mL^-1^) at 37℃ (45 min). Subsequently, the mixture was gently grinded and filtered through 100 µm cell strainer. Meanwhile, spleen was washed by PBS and lightly crushed through plunger of the syringe. Hereafter, the mixture was filtered through 100 µm cell strainer. Simultaneously, red cells were removed by 1× RBC Lysis Buffer. Finally, lung was minced into small pieces and mixed with FAC solution (PBS containing 1% serum) at 37℃ (45 min). Thereafter, the mixture was gently chopped and filtered via 100 µm cell strainer so that can gain single cell suspension. In order to analyze the level of DC, CD4 and CD8 cells, the cells were stained with FITC anti-mouse FITC Anti-Mouse CD45, PE anti-mouse CD11c, PE-Cy7 anti-mouse CD86 (DC cell), APC Anti-Mouse CD3, APC-Cyanine7 Anti-Mouse CD4, PE Anti-Mouse CD8 (CD4 and CD8) following the vendor’s protocols. Hereafter, the stained cells were analyzed by flow cytometry.

**In vivo photodynamic therapy and biosafety assessment**

The 4T1 tumor-bearing mice were divided into four groups when the tumor volume is about 100 mm^3^. For the PBS +laser and PDI(Br)_6_ NPs+laser, PBS or PDI(Br)_6_ NPs were intravenously injected with 808 nm laser irradiation for 10 min at 12 h post-injection, respectively. By contrast, the same volume of PBS and PDI(Br)_6_ NPs were intravenously injected without 808 nm laser irradiation, respectively, named PBS and PDI(Br)_6_ NPs group. In the next 18 days, the weight and tumor volume of mice were recorded every two days and calculated as follows: volume = length × width^2^/2. After that has all finished, the blood from the mice were collected into sterilized PE tubes. The whole blood samples were stored in an anticoagulant tube with EDTA and centrifuged for 15 minutes to take the supernatant. All serum samples from the different groups were acquired for kidney and liver function. In the end, the mice were sacrificed by injecting an overdose of narcotics and the major organs (heart, liver, spleen, lung, and kidney) and tumors were collected and imaged using the DSLR camera. All tumors from the different groups were harvested for antitumor efficacy assessment, and the major organs were collected for H&E staining, immu1nohistochemistry and immunofluorescence staining (CD8^+^, Ki67, TUNEL, HMGB1).4

**Blood biochemical test.**

The blood was collected and placed in a biochemical incubator (30 min), and then centrifuged at 6000 rpm (10 min). The supernatant was used to detect the following indicators including alanine aminotransferase (ALT), aspartate aminotransferase (AST), creatinine (CREA) and blood uric acid (UA).

**Statistical analysis.**

All values in the present study are expressed as mean ± s.d. unless otherwise indicated. The significance between two groups was analyzed by two-tailed Student’s t-test. For multiple normally distributed data sets with equal variances comparisons, one-way ANOVA with Tukey post-hoc test was used. P value of less than 0.05 was considered significant. ns: P > 0.05, *P < 0.05, **P < 0.01, ***P < 0.001, ****P < 0.0001) Statistical analysis was performed using SPSS.

**4. Synthetic and characterization**

**4.1. Synthesis of PDI(Br)_0_**

**Scheme S1.** The synthetic routes of PDI(Br)_0_.

PDI(Br)_0_ 3,3'-(1,3,8,10-tetraoxo-1,3,8,10-tetrahydroanthra[2,1,9-def:6,5,10-d'e'f'] diisoquinoline-2,9-diyl) bis (N-benzyl-N, N-dimethylpropan-1-aminium) was synthesized on the basis of the literature reported program with some modifications, ^[1,2]^ 1 g 3,4,9,10-perylenetetracarboxylic dianhydride and 30 mL N, N-dimethyl-1,3-propanediamine were mixed in a flask. The mixture was refluxed overnight (around 20 h), altering the color from red to dark brown. The dark brown solid was filtered directly and the filter cake was washed three times with methanol and then dried under vacuum. The brown solid precipitate was compound BADT, with a yield of 95%. ^1^H NMR (600 MHz, C_2_DF_3_O_2_) δ 8.93–8.78 (m, 8H), 4.58 (t, *J* = 6.8 Hz, 4H), 3.52 (t, *J* = 7.3 Hz, 4H), 3.21 (d, *J* = 1.2 Hz, 12H), 2.54 (p, *J* = 7.0 Hz, 4H). 0.15 g compound BADT (2,9-bis(3-(dimethylamino) propyl) anthra[2,1,9-def:6,5,10-d'e'f'] diisoquinoline-1,3,8,10(2H,9H)-tetraone) and 1.5 g benzyl bromide were mixed with 30 mL DMF. The mixture was poured into a flask heating up to 90 °C for 12 h. The red solid precipitate PDI(Br)_0_ was filtered directly and washed by acetonitrile and diethyl ether for three times. ^1^H NMR (600 MHz, DMSO-*d_6_*) δ 8.47-8.36 (m, 4H), 8.20 (t, *J* = 11.7 Hz, 4H), 7.57-7.42 (m, 10H), 4.57 (s, 4H), 4.11 (q, *J* = 12.0, 9.3 Hz, 4H), 3.47-3.38 (m, 4H), 2.98 (s, 12H), 2.26 (t, *J* = 8.4 Hz, 4H). ESI-MS: m/z =371.1752 (Calculated for C_48_H_46_N_4_O_4_^2+^: m/z = 371.1754).

**4.2. Synthesis of PDI(Br)_2_**

**Scheme S2.** The synthetic routes of PDI(Br)_2_.

Regioisomerically pure 1,7-dibromoperylene-3,4,9,10-tetracarboxylic dianhydride (3.50 g, 6.36 mmol), synthesized according to literature procedure. ^[3, 4]^ pure 1,7-dibromoperylene-3,4,9,10-tetracarboxylic dianhydride (1 g, 1.82 mmol) and 3-(Dimethylamino) propyl-1-amine (2.00 mL, 15.9 mmol) was added to the mixture, and the reaction was stirred at 65 °C for 15 h under N_2_. Propionic acid was added as a catalyst. The mixture was concentrated under reduced pressure and then precipitated into diethyl ether and subjected to centrifugation; this process was repeated three times until the supernatant appeared colorless. The product was dissolved in chloroform, washed three times with brine and once with DI water, and then dried over anhydrous Na_2_SO_4_ to obtain compound 2Br-BADT (5,12-dibromo-2,9-bis(3-(dimethylamino) propyl) anthra [2,1,9-def:6,5,10-d'e'f'] diisoquinoline-1,3,8,10(2H,9H)-tetraone). ^1^H NMR (600 MHz, Chloroform-*d*) δ 9.48 (d, *J* = 7.7 Hz, 2H), 8.92 (s, 2H), 8.70 (d, *J* = 8.0 Hz, 2H), 4.26 (t, *J* = 8.0 Hz, 4H), 2.43 (t, *J* = 7.0 Hz, 4H), 2.25 (s, 12H), 1.97 – 1.89 (m, 4H). ESI-MS: m/z: [M+H] ^+^ calculated for C_34_H_31_Br_2_N_4_O_4_: 719.0.92; found: 719.0667. 0.20 g compound 2Br-BADT and 1.5 g benzyl bromide were mixed with 30 mL EtOH/DMF. The mixture was poured into a flask heating up to 90 °C for 12 h. The red solid precipitate PDI(Br)_2_ (3,3'-(5,12-dibromo-1,3,8,10-tetraoxo-1,3,8,10-tetrahydroanthra [2,1,9-def:6,5,10-d'e'f'] diisoquinoline-2,9-diyl) bis (N-benzyl-N, N-dimethylpropan-1-aminium) was filtered directly and washed by acetonitrile and diethyl ether for three times. ^1^H NMR (600 MHz, DMSO-*d_6_*) δ 8.64 (d, *J* = 3.4 Hz, 3H), 8.62 (d, *J* = 3.7 Hz, 3H), 7.53 – 7.49 (m, 4H), 7.45 (q, *J* = 6.5 Hz, 4H), 7.42 – 7.25 (m, 2H), 4.51 (s, 3H), 4.17 – 4.11 (m, 5H), 3.43 – 3.37 (m, 4H), 3.18 – 3.15 (m, 2H), 2.92 (s, 6H), 2.24 (dt, *J* = 11.5, 6.0 Hz, 4H), 2.15 – 2.03 (m, 4H). ESI-MS: m/z =450.0871 (Calculated for C_48_H_44_Br_2_N_4_O_4_^2+^: m/z = 450.0849).

**4.3. Synthesis of PDI(Br)_4_**

**Scheme S3.** The synthetic routes of PDI(Br)_4_.

Compound A was synthesized according to the literature with slight modifications. ^[5,6]^ The compound A 1 g was weighed and dissolved in 50 mL of NMP followed by 5 mL of N N-dimethyl-1,3-propanediamine, stirred for 30 min and then 2 mL of propionic acid was added as catalyst. The crude product was purified, and 4Br-BADT (5,6,12,13-tetrabromo-2,9-bis(3-(dimethyllamino) propyl) anthra [2,1,9-def:6,5,10-d'e'f'] diisoquinoline-1,3,8,10(2H,9H)-tetraone) was collected as a red powder. ^1^H NMR (600 MHz, Chloroform-*d*) δ 8.81 (s, 4H), 4.35-4.15 (m, 4H), 2.44 (t, *J* = 7.2 Hz, 4H), 2.26 (s, 12H), 1.92 (q, *J* = 7.4 Hz, 4H). ESI-MS: m/z: [M+H] ^+^ calcd for C_34_H_29_Br_4_N_4_O_4_: 876.8881; found 876.8875. 0.15 g compound 4Br-BADT and 1 g benzyl bromide were mixed with 20 mL THF/EtOH. The mixture was poured into a flask heating up to 90 °C for 10 h. The red solid precipitate PDI(Br)_4_ 3,3'-(5,6,12,13-tetrabromo-1,3,8,10-tetraoxo-1,3,8,10-tetrahydroanthra[2,1,9-def:6,5,10-d'e'f'] diisoquinoline-2,9 diyl) bis (N-benzyl-N,N-dimethylpropan-1-aminium) was filtered directly and washed by acetonitrile and diethyl ether for three times. The yield was 85%. ^1^H NMR (600 MHz, Deuterium Oxide) δ 8.20 (s, 4H), 7.44 (s, 4H), 7.40 (s, 2H), 7.35 (d, *J* = 35.1 Hz, 4H), 4.45 (s, 2H), 4.20 (s, 2H), 2.98 (s, 4H), 2.83 (s, 8H), 2.77 (s, 2H), 2.73 (d, *J* = 2.4 Hz, 8H), 2.47 (s, 2H). ESI-MS: m/z =528.9942 (Calculated for C_48_H_42_Br_4_N_4_O_4_^2+^: m/z = 528.9944).

**4.4. Synthesis of PDI(Br)_6_**

**Scheme S4.** The synthetic routes of PDI(Br)_6_.

Compound B 1g was weighed and dissolved in 50 mL of NMP followed by 5 mL of N, N-dimethyl-1,3-propanediamine, stirred for 30 min and then 2 mL of propionic acid was added as catalyst. The crude product was purified, and 6Br-BADT was collected as a red powder. ^1^H NMR (600 MHz, Chloroform-*d*) δ 8.85 (d, *J* = 11.0 Hz, 1H), 8.80 (d, *J* = 11.7 Hz, 1H), 4.28 (tt, *J* = 11.5, 6.3 Hz, 4H), 2.49 (q, *J* = 8.0, 7.5 Hz, 4H), 2.30 (s, 12H), 1.95 (q, *J* = 7.4 Hz, 4H). ESI-MS: m/z: [M+H] ^+^ calcd for C_34_H_27_Br_6_N_4_O_4_: 1034.7071; found 1034.7116. 0.20 g compound 6Br-BADT and 1.0 g benzyl bromide were mixed with 50 mL THF/EtOH. The mixture was poured into a flask heating up to 90 °C for 10 h. The black-red solid precipitate PDI(Br)_6_ was filtered directly and washed by acetonitrile and diethyl ether for three times. ^1^H NMR (600 MHz, DMSO-*d_6_*) δ 8.98 (d, *J* = 48.9 Hz, 2H), 8.29 (s, 4H), 8.06 (s, 2H), 7.91 (s, 4H), 4.02 (s, 4H), 3.09 (t, *J* = 5.8 Hz, 12H), 2.85 (s, 4H), 2.69 (s, 4H), 1.93 (s, 4H). ESI-MS: m/z =607.9054 (Calculated for: C_48_H_40_Br_6_N_4_O_4_^2+^: m/z = 607.9039).

**5. Supplementary figures**

**Figure S1.** ^1^H NMR spectrum of BADT in C_2_DF_3_O_2_.

**Fig****ure S2.** ^1^H NMR spectrum of PDI(Br)_0_ in DMSO-*d_6._*

**Figure S3.** HR-MS spectrum of PDI(Br)_0_.

**Figure S4.** ^1^H NMR spectrum of 2Br-BADT in CDCl_3_.

**Figure S5.** ^1^H NMR spectrum of PDI(Br)_2_ in DMSO-*d6.*

**Figure S6.** HR-MS spectrum of 2Br-BADT.

**Figure S7.** HR-MS spectrum of PDI(Br)_2_.

**Figure S8.** ^1^H NMR spectrum of 4Br-BADT in CDCl_3._

*__*

**Figure S9.** ^1^H NMR of compound PDI(Br)_4_ in D_2_O.

**Figure S10.** HR-MS spectrum of 4Br-BADT.

**Figure S11.** HR-MS spectrum of PDI(Br)_4_.

**Figure S12.** ^1^H NMR spectrum of 6Br-BADT in CDCl_3_.

**Figure S13.** ^1^H NMR spectrum of PDI(Br)_6_ in DMSO-*d_6._*

**Figure S14.** HR-MS spectrum of 6Br-BADT.

**Figure S15.** HR-MS spectrum of PDI(Br)_6_.

**Figure S16.** Zeta potential analysis of PDI(Br)_0/2/4/6_ and PDI(Br)_0/2/4/6_ NPs respectively.

**Figure S17.** DLS analysis of PDI(Br)_0/2/4_ NPs, respectively.

**Figure S18.** Size changes of PDI(Br)_0/2/4/6_ NPs in PBS solution supplemented with 10% fetal bovine serum during a period of time storage at room temperature, respectively. (the results are presented as mean ± SD, n = 3).

**Figure S19.** (a) Normalized absorption spectrum of PDI(Br)_0/2/4/6_ NPs and (b) fluorescence spectrum (excitation wavelength 525 nm), respectively.

**Figure S20.** The fluorescence spectrum changes of PDI(Br)_4/6_ NPs with the addition of reducing agents (Na_2_S_2_O_3_, Na_2_S_2_O_4_), respectively.

**Figure S21.** The absorption spectrum of [PDI(Br)_0/2/4/6_] NPs^•−^ were produced in the presence of different amounts of Na_2_S_2_O_4_, respectively.

**Figure S22.** The absorption spectrum of [PDI(Br)_0/2/4/6_] NPs^•−^ were produced in the presence of different amounts of Na_2_S_2_O_3_, respectively.

**Figure S23.** Cyclic voltammograms of PDI(Br)_0/2/4/6_ in solutions.

Conditions:

The auxiliary electrode is Platinum wire electrode.

The reference electrode is Ag/AgCl.

The working electrode is glassy carbon.

The supporting electrolyte is 50.0 mM NaCl solution.

The temperature is 298 K. The scan rate is 10 mV/s.

The concentration is 0.12 mM.

**
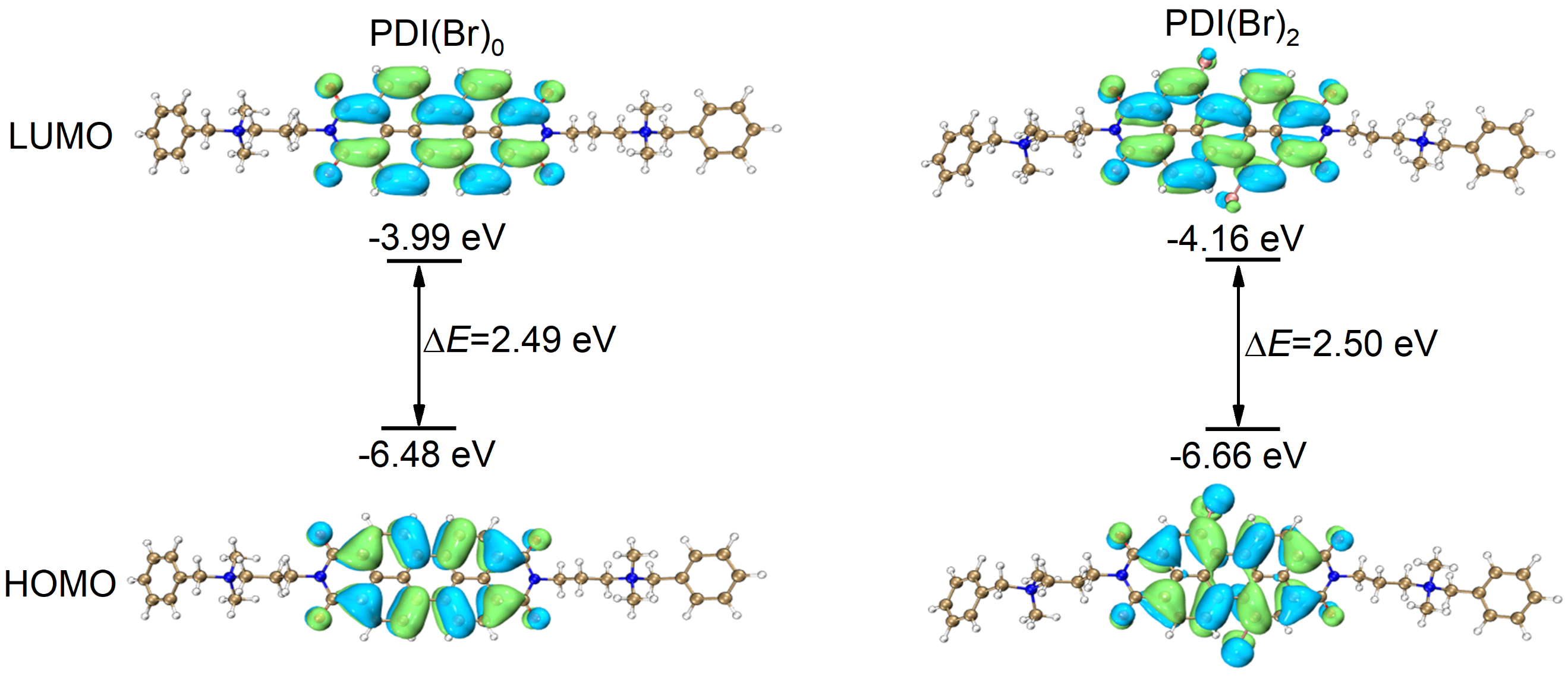
**

**Figure S24.** Calculated frontier molecular orbitals and energy levels of the PDI(Br)_0/2_.

**
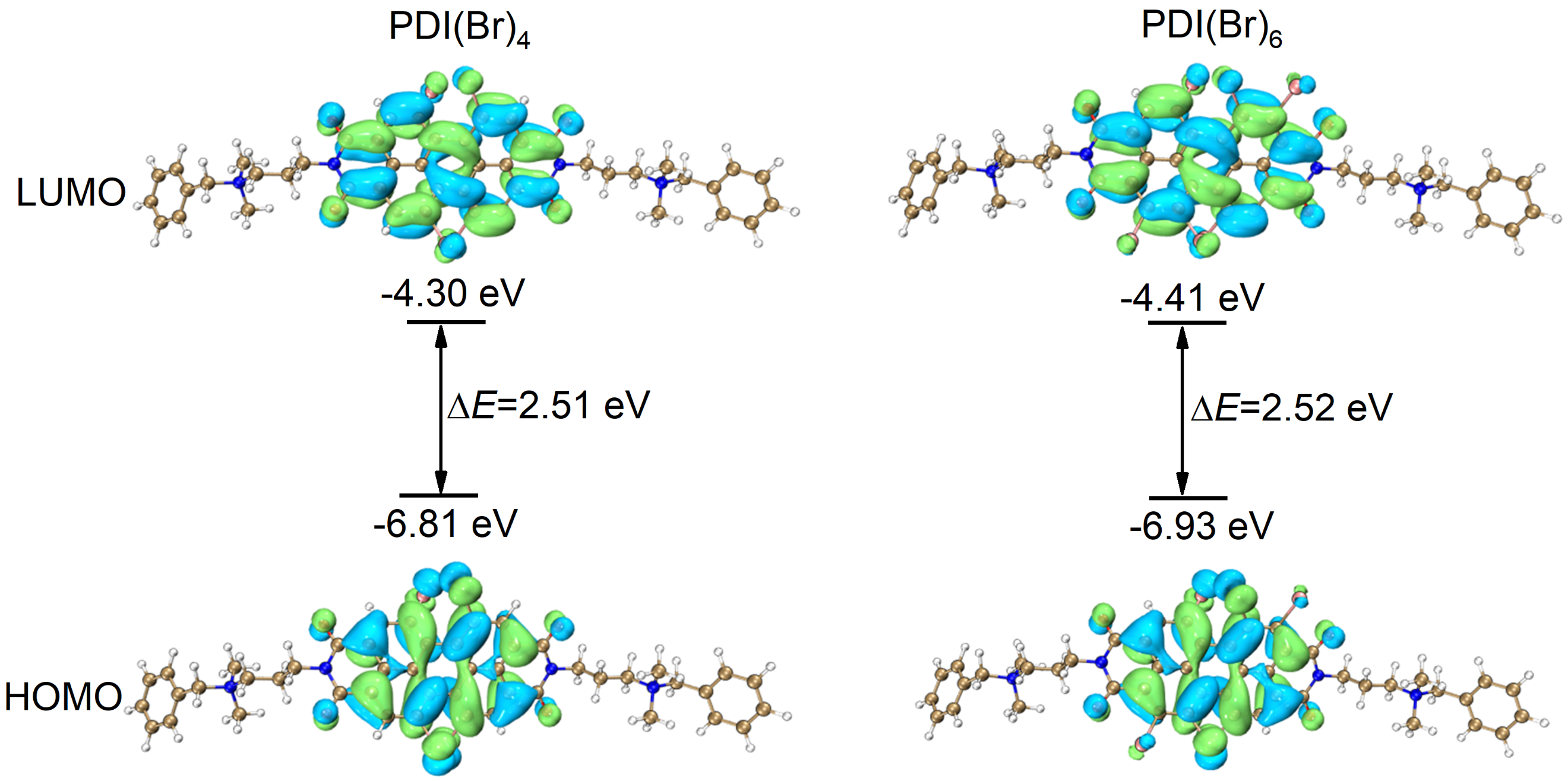
**

**Figure S25.** Calculated frontier molecular orbitals and energy levels of the PDI(Br)_4/6_.

**Figure S26.** The NIR-II fluorescence spectrum of PDI(Br)_0/2/4/6_ NPs in the presence of different amounts of Na_2_S_2_O_4_. (*E_x_* = 808 nm).

**Figure S27.** The NIR-II fluorescence spectrum of PDI(Br)_0/2/4/6_ NPs in the presence of different amounts of Na_2_S_2_O_3_. (*E_x_* = 808 nm).

**Figure S28.** The stability of [PDI(Br)_4_] NPs^•−^ under hypoxia conditions.

**Figure S29.** The stability of [PDI(Br)_4_] NPs^•−^ under different pH conditions, pH=5.0, pH=6.0, pH=6.5, pH=7.4 respectively.

**Figure S30.** The stability of [PDI(Br)_6_] NPs^•−^ under different pH conditions, pH=5.0, pH=6.0, pH=6.5, pH=7.4, respectively.

**Figure S31.** The stability of [PDI(Br)_4/6_] NPs^•−^ with 10% fetal bovine serum (FBS), respectively.

**Figure S32.** (a, b) Photostability test of [PDI(Br)_4/6_] NPs^•−^, respectively. (808 nm laser irradiation, 0.6 W/cm^2^). (c, d) Absorption spectrum of [PDI(Br)_4/6_] NPs^•−^ and ICG before and after 6 min of 808 nm laser irradiation (0.6 W/cm^2^), respectively.

**Figure S33.** Photothermal stability test of [PDI(Br)_4/6_] NPs^•−^ with multiple cycles of laser exposure (808 nm, 0.6 W/ cm^2^), respectively.

**Figure S34.** The dihedral angles of PDI(Br)_0/2/4/6_, respectively.

**Figure S35.** The dihedral angles of [PDI(Br)_0/2/4/6_] ^•−^, respectively.

**Figure S36.** The SUMO frontier molecular orbitals and energy levels of [PDI(Br)_0/2/4/6_] ^•−^, respectively.

**Figure S37.** The changing amount of generated radical anions by adding Na_2_S_2_O_3_ to PDI(Br)_4/6_ NPs solutions. The excess Na_2_S_2_O_3_ was removed by ultrafiltration centrifugation method.


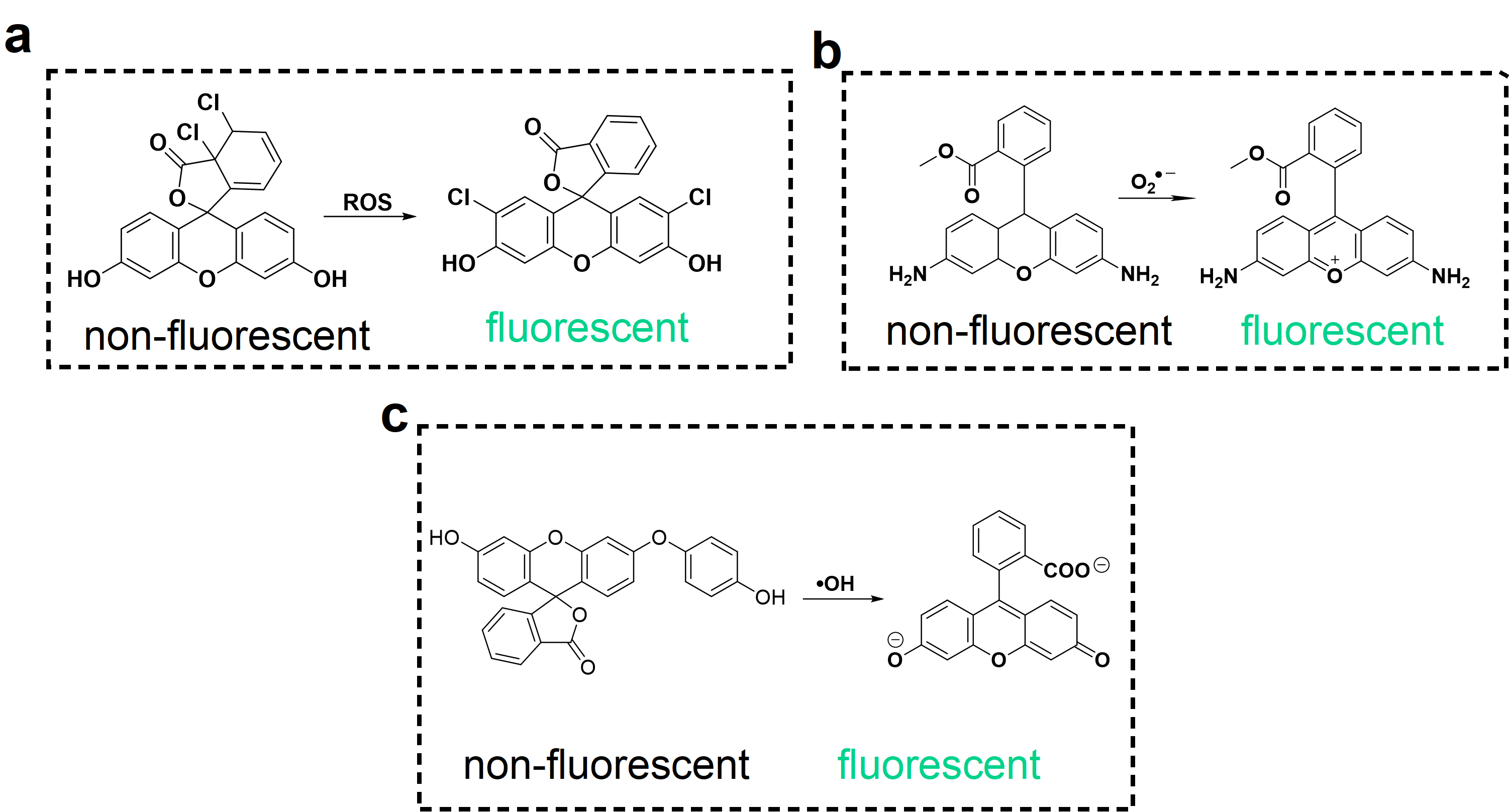


**Figure S38.** (a) Mechanism of DCFH as an indicator for ROS superoxide radical generation. (b) Mechanism of DHR123 as an indicator for superoxide radical generation. ^[7]^ (c) Mechanism of HPF as an indicator for superoxide radical generation. ^[8]^

**Figure S39.** Fluorescence intensity changes of DCFH for total ROS detection. (a) [PDI(Br)_4_] NPs^•－^, (b) [PDI(Br)_6_] NPs^•－^, (c) DCFH only with increasing irradiation time (0-9 min) upon 808 nm laser irradiation (power density :0.30 W cm^-2^).

**Figure S40.** Fluorescence intensity changes of DCFH for endogenous ROS detection. (a) [PDI(Br)_4_] NPs^•－^, (b) [PDI(Br)_6_] NPs^•－^, (c) DCFH only. (d) Comparison of the ROS generation capacity of PBS, [PDI(Br)_4/6_] NPs^•－^with or without laser irradiation.

**Figure S41.** Fluorescence intensity changes of DHR123 for O_2_^•－^ detection. (a) [PDI(Br)_4_] NPs^•－^, (b) [PDI(Br)_6_] NPs^•－^, (c) DHR123 only with increasing irradiation time (0-9 min) upon 808 nm laser irradiation (power density : 0.3 W cm^-2^). (d) Comparison of the O_2_^•－^ generation. GI: [PDI(Br)_6_] NPs^•－^+laser, GII: [PDI(Br)_4_] NPs^•－^+laser, GIII: PBS+laser.

**Figure S42.** O_2_^•−^ generation of [PDI(Br)_4/6_] NPs^•－^ under hypoxic conditions with increasing irradiation time (0-9 min) upon 808 nm laser irradiation (power density :0.3 W cm^-2^).

**Figure S43.** Fluorescence intensity changes of DHR123 for endogenous O_2_^•－^ detection. (a) [PDI(Br)_4_] NPs^•－^, (b) [PDI(Br)_6_] NPs^•－^, (c) DHR123 only. (d) Comparison of the endogenous O_2_^•－^. GI: [PDI(Br)_6_] NPs^•－^, GII: [PDI(Br)_4_] NPs^•－^, GIII: PBS.

**Figure S44.** Fluorescence intensity changes for •OH detection. (a) [PDI(Br)_4_] NPs^•－^, (b) [PDI(Br)_6_] NPs^•－^ (c) HPF probe upon 808 nm laser irradiation (0-9 min) (power density :0.3 W cm^-2^). (d) The comparison of •OH. 200 μM of H_2_O_2_ was added to all test solutions. GI: [PDI(Br)_6_] NPs^•－^+laser, GII: [PDI(Br)_4_] NPs^•－^+laser, GIII: PBS+laser.

**Figure S45.** Fluorescence intensity changes for endogenous •OH detection. (a) [PDI(Br)_4_] NPs^•－^, (b) [PDI(Br)_6_] NPs^•－^(c) HPF only. (d) Comparison of the endogenous •OH generation. 200 μM of H_2_O_2_ was added to all test solutions. GI: [PDI(Br)_6_] NPs^•－^, GII: [PDI(Br)_4_] NPs^•－^, GIII: PBS.

**Fig****ure S46.** ^1^O_2_ generation of [PDI(Br)_4/6_] NPs^•－^ upon 808 nm laser irradiation (0-9 min) (power density :0.3 W cm^-2^), respectively.

**Figure S47.** Electrostatic potential-mapped electron density diagrams of PDI(Br)_0/2_, respectively.

**Figure S48.** Electrostatic potential-mapped electron density diagrams of [PDI(Br)_0/2_]**^•−^** respectively.


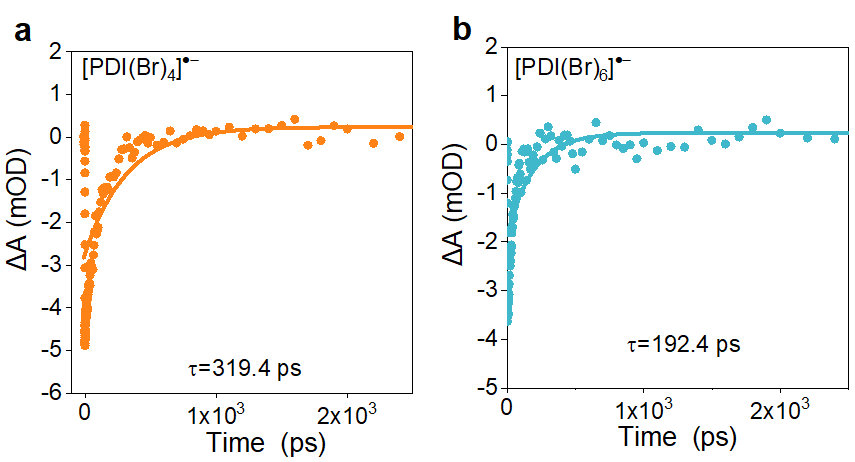


**Figure S49.** Transient absorption kinetics monitored at 921 nm for [PDI(Br)_4_]**^•−^**, 942 nm for [PDI(Br)_6_]**^•−^** with an overlaid fit to the single exponential function, respectively.

**Figure S50.** The NIR-II fluorescence intensity of normal cells and tumor cells, respectively (1: NIH 373 cells, 2: L929 cells, 3: 4T1 cells, 4: B16F10 cells, 5: A20 cells, 6: H22 cells).

**Figure S51.** The stability test of [PDI(Br)_0/2/4/6_]**^•−^** generated from the four kinds of PDI(Br)_n_ NPs incubating with 4T1 tumor cells.

**Figure S52.** Spin density distribution of [PDI(Br)_0/2_]**^•−^** respectively.

**Figure S53.** Spin density distribution of [PDI(Br)_4/6_]**^•−^** respectively.

**Figure S54.** Flow cytometry analysis of total ROS in 4T1 cells with or without the treatment of PDI(Br)_6_ NPs with or without laser irradiation.

**Figure S55.** Representative fluorescence images of H_2_O_2_ in 4T1 cancer cells after various treatments in hypoxia environment. Scale bar =50 μm.

**Figure S56.** Representative fluorescence images and flow cytometry analysis of •OH of 4T1 cells after various treatments in hypoxia. Scale bar =50 μm.


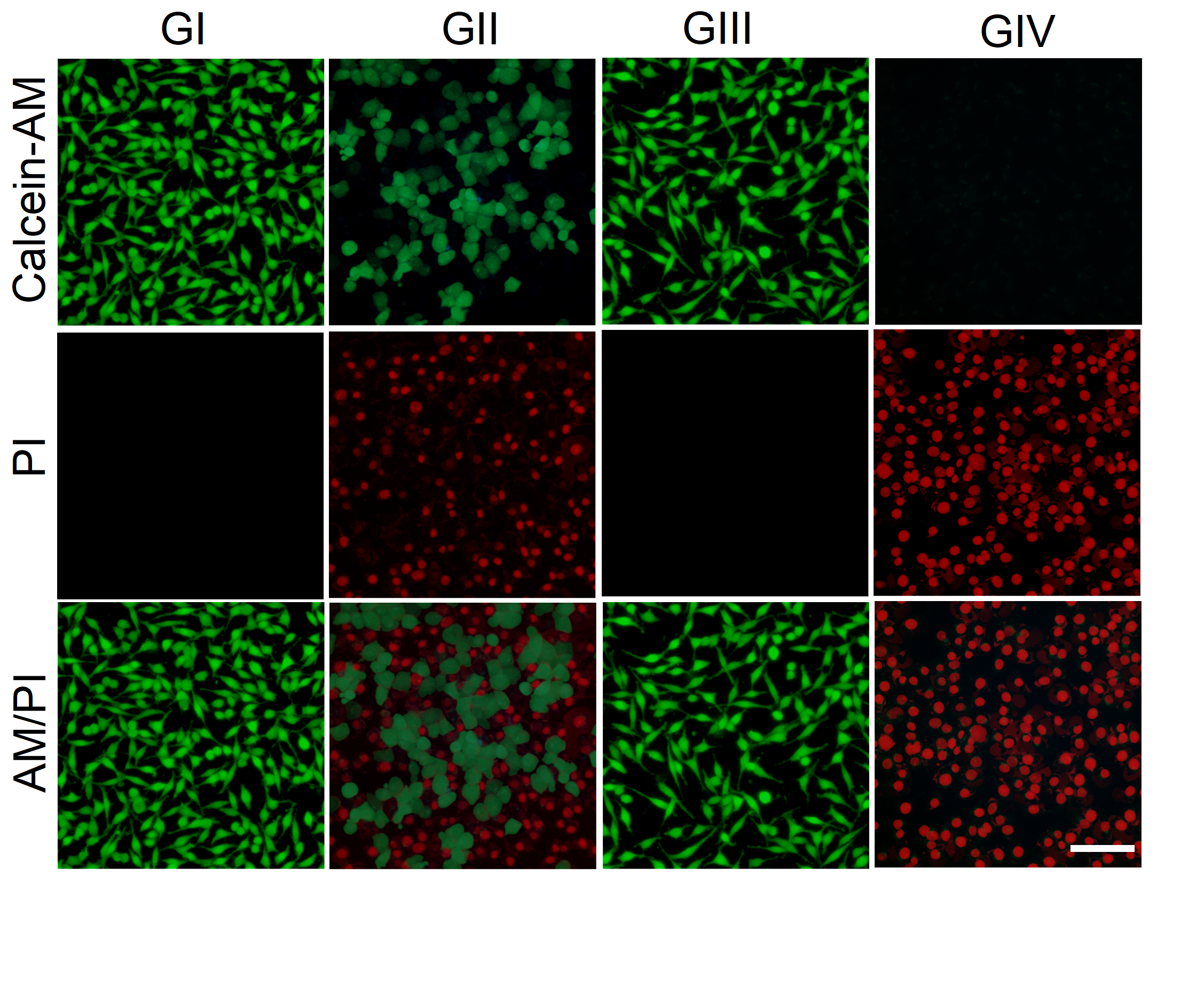


**Figure S57.** Live and dead cells imaging of 4T1 cancer cells after various treatments. GI: PBS, GII: PDI(Br)_6_ NPs, GIII: PBS+laser, GIV: PDI(Br)_6_ NPs+laser. Scale bar =50 μm.

**Figure S58.** Detection of ATP secreted into the medium of 4T1 cancer cells after various treatments. Data are presented as mean ± SD (n = 3).

**Figure S59.** Representative CLSM images of the HMGB1 release on 4T1 cancer cells after various treatments. Scale bar: 40 μm.

**Figure S60.** Representative CLSM images of the CRT exposure on 4T1 cancer cells after various treatments. Scale bar: 40 μm.

**Figure S61.** (a) Experimental outline recording the NIR-II FLI steps and procedures in 4T1 tumor-bearing mice. (b, c) Representative tumor specific NIR-II FLI and their corresponding intensities of 4T1 tumor bearing mice at different time points after intravenous injection of PDI(Br)_4_ NPs, respectively. Data are presented as mean ± SD (n = 3).

**Figure S62.** The NIR-II FLI and the corresponding fluorescence intensities of the major organs and tumor from mice after 36 h post-injection of PDI(Br)_4_ NPs. Data are presented as mean ± SD (n = 3).

**Figure S63.** (a) Experimental outline recording the NIR-II FLI steps and procedures in 4T1 tumor-bearing mice. (b) Representative tumor specific NIR-II FLI of tumor mice at different time points after intravenous injection of PDI(Br)_0_ NPs. (c, d) The images of the major organs and tumor in bright-field and NIR-II channel after systemic injection of PDI(Br)_0_ NPs.

**Figure S64.** (a) Experimental outline recording the NIR-II FLI steps and procedures in healthy mice. (b) Representative NIR-II FLI of healthy mice at different time points after intravenous injection of PDI(Br)_6_ NPs. (c, d) The images of the major organs (heart, liver, spleen, lung, kidney intenstines) in bright-field and NIR-II channel after systemic injection of PDI(Br)_6_ NPs.

**Figure S65.** (a) Experimental outline recording the NIR-II FLI steps and procedures in healthy mice. (b) Representative NIR-II FLI of healthy mice at different time points after intravenous injection of PDI(Br)_4_ NPs. (c, d) The images of the major organs (heart, liver, spleen, lung, kidney intenstines) in bright-field and NIR-II channel after systemic injection of PDI(Br)_4_ NPs.

**Figure S66.** The tumor volume changes of 4T1 tumor-bearing mice after different treatments. (mean± SD, n = 5).

**Figure S67.** H&E staining analysis of major organs of 4T1 tumor-bearing mice after various treatment. GI: PBS, GII: PDI(Br)_6_ NPs, GIII: PBS+laser, GIV: PDI(Br)_6_ NPs+laser. Scale bars: 100 μm.

**Figure S68.** Blood biochemistry test of 4T1 tumor-bearing mice after various treatment. (a, b) hepatic function markers and (c, d) renal function markers. GI: PBS, GII: PDI(Br)_6_ NPs, GIII: PBS+laser, GIV: PDI(Br)_6_ NPs+laser.

**6. Supplementary references**

[1] Y. Jiao, K. Liu, G. T. Wang, Y. P. Wang, X. Zhang, *Chem. Sci.* **2015**, *7*, 6.

[2] H. Wang, K. F. Xue, Y. C. Yang, H. Hu, J. F. Xu, X. Zhang, *J. Am. Chem. Soc.* **2022**, *144*, 2360.

[3] S. Sengupta, R. K. Dubey, R. W. M. Hoek, S. P. P. van Eeden, D. Deniz Gunba, F. C. Grozema, E. J. R. Sudhölter, W. F. Jager, *J. Org. Chem.* **2014**, *79*, 6655.

[4] A. Alvino， M. Franceschin， C. Cefaro，S. Borioni，A. Bianco, *Tetrahedron* **2007**, *63*, 7858.

[5] J. Zhang, W. Ma, H. F. Luo, K. X. Zhang, J. Q. Lv, L. Z. Jiang, Y. L. Huang, J. B. Song, Z. Yang, W. Huang, *Adv. Healthcare Mater.* **2024**, *13*, 2303175.

[6] M. Cole， M. Sheri， C. Bielicki， T. Emrick, *Macromolecules* **2017**, *50*, 7535.

[7] J. H. Zou, Z. Li, Zhu, Y. Y. C. Tao, Q. You, F. F. Cao, Q. H. Wu, M. Wu, J. J. Cheng, J. W. Zhu, X. Y. Chen, *Bioact. Mater.* **2024**, *34*, 414.

[8] M. L. Li, T. Xiong, J. J. Du, R. S. Tian, M. Xiao, L. Y. Guo, S. R. Long, J. L. Fan, W. Sun, K. Shao, X. Z. Song, J. W. Foley, X. J. Peng *J. Am. Chem. Soc.* **2019**, *141*, 2695.
